# Muscle transcriptome analysis identifies genes involved in ciliogenesis and the molecular cascade associated with intramuscular fat content in Large White heavy pigs

**DOI:** 10.1371/journal.pone.0233372

**Published:** 2020-05-19

**Authors:** Martina Zappaterra, Silvia Gioiosa, Giovanni Chillemi, Paolo Zambonelli, Roberta Davoli

**Affiliations:** 1 Department of Agricultural and Food Sciences (DISTAL), Division of Animal Science, University of Bologna, Bologna, Italy; 2 Super Computing Applications and Innovation Department (SCAI), CINECA, Rome, Italy; 3 Department for Innovation in Biological, Agro-food and Forest systems (DIBAF), University of Tuscia, Viterbo, Italy; 4 Institute of Biomembranes, Bioenergetics and Molecular Biotechnologies (IBIOM), CNR, Bari, Italy; 5 Interdepartmental Centre of Agri-food Industrial Research (CIRI-AGRO), University of Bologna, Cesena, Italy; INIA, SPAIN

## Abstract

Intramuscular fat content (IMF) is a complex trait influencing the technological and sensorial features of meat products and determining pork quality. Thus, we aimed at analyzing through RNA-sequencing the *Semimembranosus* muscle transcriptome of Italian Large White pigs to study the gene networks associated with IMF deposition. Two groups of samples were used; each one was composed of six unrelated pigs with extreme and divergent IMF content (0.67 ± 0.09% in low IMF *vs*. 6.81 ± 1.17% in high IMF groups) that were chosen from 950 purebred individuals. Paired-end RNA sequences were aligned to *Sus scrofa* genome assembly 11.1 and gene counts were analyzed using WGCNA and DeSeq2 packages in R environment. Interestingly, among the 58 differentially expressed genes (DEGs), several were related to primary cilia organelles (such as *Lebercilin 5* gene), in addition to the genes involved in the regulation of cell differentiation, in the control of RNA-processing, and G-protein and ERK signaling pathways. Together with cilia-related genes, we also found in high IMF pigs an over-expression of the *Fibroblast Growth Factor 2* (*FGF2*) gene, which in other animal species was found to be a regulator of ciliogenesis. Four WGCNA gene modules resulted significantly associated with IMF deposition: grey60 (*P* = 0.003), darkturquoise (*P* = 0.022), skyblue1 (*P* = 0.022), and lavenderblush3 (*P* = 0.030). The genes in the significant modules confirmed the results obtained for the DEGs, and the analysis with “cytoHubba” indicated genes controlling RNA splicing and cell differentiation as hub genes. Among the complex molecular processes affecting muscle fat depots, genes involved in primary cilia may have an important role, and the transcriptional reprogramming observed in high IMF pigs may be related to an FGF-related molecular cascade and to ciliogenesis, which in the literature have been associated with fibro-adipogenic precursor differentiation.

## Introduction

Pork meat represents one of the main sources of protein and fat for humans, accounting for about 30% of meat consumption worldwide [1]. Pork eating quality, a food property that encompasses taste, flavor, juiciness, and tenderness, is affected, among others, by meat intramuscular fat (IMF) content. Higher contents of IMF are generally regarded as exerting a positive effect on meat quality features, although there is no complete agreement in the literature [2, 3]. Several studies reported that meat with a level of IMF below 2.2%-2.5% is associated with detrimental palatability [3, 4]. Anyway, due to the negative genetic correlation linking IMF deposition with carcass weight and lean percentage [5], the selection carried out by the swine industry prioritizing production efficiency and increased lean mass growth caused a consistent decrease in IMF depots [6]. IMF is a heritable trait in pigs [5, 7] and genetic selection is a promising approach to increase IMF without negatively affecting production efficiency. However, this strategy is confounded by the existence of a high number of Quantitative Trait Loci (QTLs) associated with IMF content [7, 8] and by the presence of negative associations with lean mass deposition [5]. Although the differentiation of preadipocytes into adipocytes starts in embryos and continues immediately after birth, this differentiation process slows down during the growth of the animal [9]. Together with the number of adipocytes (hyperplasia), IMF content in meat is also determined by the adipocyte size (hypertrophia) [10]. Even though the number of adipocytes interspersed in the muscle may vary among pig breeds [11], adiposity in the pig is mainly due to adipocyte hypertrophy. Adipocyte hypertrophy is caused by both genetics and environmental effects and consists of an accumulation of triglycerides in mature adipocyte as a result of a metabolism shifted towards lipogenesis [11, 12]. The complexity of the metabolic processes taking place in IMF adipocytes has been described in pigs [13] and in other animal species, where several studies suggested for IMF adipocytes different roles compared with subcutaneous and visceral adipocytes [14, 15]. Furthermore, the important role of muscle-interspersed adipocytes in muscle energy metabolism has been highlighted by the increasing evidence of the involvement of IMF in the modulation of cardiovascular risk factors [16] and insulin resistance [17], and in the existence of a muscle-to-fat “crosstalk” mediated by biologically active molecules such as adipokines and myokines [18, 19].

In this scenario, the investigation of the molecular patterns related to IMF deposition may provide new information useful for a more efficient selection aimed at increasing IMF in pork and pork products quality without negatively affecting lean mass deposition. This objective may also be of interest considering the increasing evidence linking this fat depot with some human metabolism-related diseases. Previous transcriptome studies using RNA-seq have revealed relevant results about the gene expression patterns and networks underlying IMF at different ages, breeds, and muscles [20–23]. Ayuso et al. [24] compared the *Biceps femoris* transcriptome between Iberian and Iberian x Duroc pigs, identifying as differentially expressed genes (DEGs) related to adipogenesis, lipid metabolism, and myogenesis, thus suggesting that differences in IMF and meat quality between these two genetic types may be ascribed to genes involved in these pathways. On the whole, the investigations of the muscle gene expression profiles identified DEGs involved in lipid metabolism [23, 25–27], myogenesis [23, 25] and cell proliferation [25]. Despite the increasing number of transcriptomic studies aimed at dissecting IMF deposition, the identification of major genes and the comprehension of the molecular cascade events related to this trait remain mostly unknown.

To the best of our knowledge, the scientific literature is still lacking studies analyzing changes in the transcriptome and regulatory factors associated with divergent IMF deposition in Large White heavy pigs intended for the production of Protected Designation of Origin (PDO) high-quality dry-cured hams. For these specific productions, *Semimembranosus* muscle (SM) composition is of primary importance, since it affects the sensory and nutritional quality of high-quality dry-cured hams. The pig genetic types used for this high economic-value production are characterized by higher live weight and slaughter age, characteristics that can influence the maturation stage of the muscle-interspersed adipocytes [28–30].

The present study was conceived with the objectives of i) evaluating in the SM the gene expression differences between two groups of Italian Large White purebred heavy pigs divergent for IMF content and identifying the pathways in which the differentially expressed genes are involved; ii) investigating the gene co-expression patterns related to the divergent deposition of IMF and interpreting the possible molecular mechanisms related to the variability noticed for this trait.

## Materials and methods

### Ethics approval

Sampling occurred with the permission of the Italian National Association of Pig Breeders (Associazione Nazionale Allevatori Suini, ANAS, http://www.anas.it). Animal care and slaughter of the animals used in this study were performed in compliance with the European rules (Council Regulation (EC) No. 1/2005 and Council Regulation (EC) No. 1099/2009) on the protection of animals during transport and related operations and at the time of the slaughtering. All slaughter procedures were monitored by the veterinary team appointed by the Italian Ministry of Health. All procedures were performed within the ANAS routine care and did not require the approval of an ethics committee.

### Sampling and phenotypes

Twelve individuals were chosen from a purebred population of 950 sib‐tested Italian Large White pigs already described in Davoli et al. [5]. Briefly, the pigs were from the ANAS national sib testing selection program. Pigs entered the testing station at about 30 kg live weight and were reared in the same controlled environmental conditions. During the testing period, pigs were kept separated and fed the same finishing diet at a *quasi ad libitum* feeding level (i.e. about 60% of pigs were able to ingest the entire supplied ration) until an average final live weight of about 150 kg at about eight months of age. At the end of the tests, animals were transported to a commercial abattoir located about 25 km from the test station according to Council Rule (EC) No 1/2005 on the protection of animals during transport and related operations. At the slaughterhouse, the pigs were electrically stunned and bled in a lying position in agreement with Council Regulation (EC) No 1099/2009 on the protection of animals at the time of the slaughtering. All slaughter procedures were monitored by the veterinary team appointed by the Italian Ministry of Health. After slaughter, SM samples were collected from the 950 ILW pigs, immediately frozen in liquid nitrogen and stored at −80°C in a deep freezer until further analysis. For all the gathered samples, IMF was determined by extraction with petroleum ether from 1 g of SM using an XT15 Ankom apparatus (Ankom, Macedon, NY, USA), according to Official Procedure AOCS Am 5–04 [31]. IMF was determined in % (g of IMF on 100 g of muscle tissue). Basing on the values of IMF % measured on the whole population, two divergent groups of six animals each were chosen for the transcriptome study. The two groups were composed of pigs displaying extreme and divergent contents of IMF, and they will be referred from here on as low IMF group (i.e. 6 samples with 0.51% ≤ IMF ≤ 0.74%; μ = 0.67 ± 0.09%) and high IMF group (i.e. 6 samples with 5.87% ≤ IMF ≤ 8.64%; μ = 6.81 ± 1.17%). The chosen samples were slaughtered in nine different days, balancing each group for sex and avoiding full and half-sibs.

### RNA extraction, library preparation, and sequencing

Total RNA was extracted with TRIzol Reagent (Invitrogen, Thermo Fisher Scientific, Waltham, MA, US) following the manufacturer's instruction. The extracted RNA samples were then quantified using a NanoDrop 1000 spectrophotometer (Thermo Fisher Scientific, Waltham, MA, US), and the RNA quality and integrity were assessed using an Agilent 2100 BioAnalyzer (Agilent Technologies, Santa Clara, CA, US). RNA integrity values (RIN) ranged between 7 and 8.5. Stranded total RNA libraries were prepared using the TruSeq Stranded mRNA Library Prep kit (Illumina Inc., San Diego, CA, US) following the manufacturer's suggested protocol. A paired-end sequencing strategy was chosen, in which short reads are extracted from both ends of long DNA fragments through ultra-high-throughput sequencing. The libraries were tagged, and pairs of libraries were run on a single lane of an Illumina HiSeq2500 (Illumina Inc., San Diego, CA, US). Paired-end reads of 100 bp were generated and the raw sequence data have been deposited in the Gene Expression Omnibus (GEO) expression database under the accession number: GSE144780.

### Mapping and assembly of the reads

Raw reads were obtained in FASTQ format and were quality-assessed using FastQC program (retrieved from URL: http://www.bioinformatics.babraham.ac.uk/projects/fastqc/). The quality was measured according to sequence-read lengths and base-coverage, nucleotide contributions and base ambiguities, quality scores and over-represented sequences and all samples passed the QC parameters. Terminal low-quality bases and adaptor sequences were trimmed out using Trimmomatic utility [32]. Clean reads were aligned against Ensembl reference genome *Sus scrofa* v. 11.1 (retrieved from URL: http://igenomes.illumina.com.s3-website-us-east-1.amazonaws.com/Sus_scrofa/Ensembl/Sscrofa11.1/Sus_scrofa_Ensembl_Sscrofa11.1.tar.gz) using the splice-aware read mapper HiSat2 [33].

### Differential expression analysis and Gene Ontology enrichment analysis

BAM files obtained from the read alignment were further processed with StringTie [34] to assemble known transcripts. HTSeq version 0.6.1 [35] was then used to quantify the reads and obtain the file with the gene counts. The differential gene expression analysis was carried out in the R environment [36] with the “DESeq2” package [37] that offers a method for gene-level analysis of RNA-seq data. Genes that were not expressed were filtered out and the expression counts of the remaining genes were transformed using *regularized-logarithm transformation* or *rlog* [37]. The two-group comparison was performed by considering only the group since the two groups were balanced for the numbers of gilts and barrows (3 gilts and 3 barrows per group) and the hot carcass weight (Table 1). DEGs were identified setting as selection parameters an absolute Log_2_ (Fold Change) (Log_2_FC) value greater than or equal to 0.58 (|Fold Change| ≥ 1.5) and a False Discovery Rate adjusted *P*-value less than or equal to 0.05 (q ≤ 0.05). Genes showing a q value comprised between 0.10 and 0.05 were considered as genes showing a difference with a trend towards significance. Fold change was calculated as the ratio of the normalized expression levels of a gene between low IMF and high IMF groups. The R package "mygene" [38] was used to match the Ensembl Gene ID to the corresponding official Gene Symbol, "org.Ss.eg.db" [39] from Bioconductor was used for genome-wide annotation and the packages "clusterProfiler" [40] and "AnnotationHub" [41] were used to compute Gene Ontologies (GOs). GO enrichment analyses of DEGs were performed using the GO terms of molecular functions (MF), biological processes (BP) and cellular components (CC). *P*-values were adjusted with False Discovery rate method and adjusted *P*-values ≤ 0.05 were considered significant.

**Table 1:** Sex, intramuscular fat (IMF) % and group membership of the used pig samples.

Sample	Sex	Carcass weight (kg)	IMF %	Group
1	Gilt	132	8.64	High IMF
2	Gilt	120	7.82	High IMF
3	Gilt	105	6.65	High IMF
4	Barrow	126	5.99	High IMF
5	Barrow	120	5.89	High IMF
6	Barrow	120	5.87	High IMF
7	Barrow	120	0.74	Low IMF
8	Barrow	124	0.73	Low IMF
9	Gilt	119	0.71	Low IMF
10	Gilt	110	0.67	Low IMF
11	Barrow	127	0.64	Low IMF
12	Gilt	116	0.51	Low IMF

### Validation by RT-qPCR

RNA samples from the 12 selected animals were used to carry out the technical validation of some of the DEGs. After DNAse treatment (TURBO DNA-free^TM^, Ambion, Applied Biosystems), 1 μg of total RNA was reverse transcribed using the iScript cDNA Synthesis kit (Bio-Rad Laboratories, Hercules, CA) according to the manufacturer’s instructions. Real-time quantitative PCR (RT-qPCR) was performed on Rotor Gene 6000 (Corbett Life Science, Concorde, New South Wales, Australia) using 5 μL of iTaq Universal SYBR Green Supermix (Bio-Rad Laboratories, Hercules, CA), 5 pmol of each primer, 2 μL of cDNA template diluted 1:10 in nuclease-free water and then was made up to the total volume of 10 μL with water. Rotor Gene 6000 protocol was performed using a two-step amplification with cycles constituted by a denaturation phase at 95°C for 5 seconds, followed by an annealing-extension step for 20 seconds using specific annealing temperatures for each primer couple (S1 Table). Primers were designed using Primer3Plus (URL: http://www.bioinformatics.nl/cgi-bin/primer3plus/primer3plus.cgi) and Primer-BLAST (URL: https://www.ncbi.nlm.nih.gov/tools/primer-blast/) online software, or were obtained from previous researches. The complete list of primer sequences and the relative annealing-extension temperatures are shown in the S1 Table. The samples were first used to assess the expression level of 3 normalizing genes that were already tested in our previous researches: *Beta-2-microglobulin* (*B2M*) [42], *Ribosomal Protein S18* (*RPS18*) and *Ribosomal Protein L32* (*RPL32*) [43]. Three replicates for each sample were performed (2 replicates in the same RT-qPCR run and a third replicate in a separate run) and the maximum variation coefficient between replicates was set at 0.2. RT-qPCR runs were considered only if amplification efficiencies were high (slopes < -3.25 and R2 ≥ 0.99). These values were automatically calculated by Rotor Gene 6000 using dynamic tube normalization and noise slope correction. After the amplification stage, dissociation curves were obtained for each replicate with the Melt step. Single peaks in the dissociation curves confirmed the specific amplification of the genes. For each sample, the relative quantification of a target gene was calculated by dividing the mean obtained for the triplicate measurements of the target gene expression by the geometric mean of the three normalizing gene expressions. The expression levels were calculated using the standard curve methods, according to Pfaffl [44]. Standard curves were obtained amplifying 12 progressive dilutions (from 10^9^ to 25 molecules/μl) of a cDNA sample at a known concentration, obtained by PCR, as described in Davoli et al. [45]. The five target genes were chosen among the DEGs for their functional role and/or their average-to-low absolute Log_2_FC values. The technical validation was performed by calculating the Pearson correlation coefficient and the coefficient of determination (R^2^) between the Log_2_FC of the expression values from RNA-seq data (FPKM) and the Log_2_FC of the normalized gene expression data obtained with RT-qPCR. These statistics were performed using the “*stats*” package in the R environment [36] and basic Excel functions.

### Weighted correlation network analysis

To identify strongly co-expressed genes involved in IMF deposition, we employed a co-expression analysis approach using the “WGCNA” package [46] in the R environment [36]. Scale-free undirected co-expression networks were built, and modules of genes significantly associated (*P*-value ≤ 0.05) with IMF variability were detected and further analyzed.

Pearson’s correlations between each gene were calculated to build an adjacency matrix. Subsequently, the matrix was raised by a Soft threshold Power (β) of 6, which was found to be an appropriate value by the function *pickSoftThreshold()* to reach a scale-free topology index (*R*^2^) of at least 0.70 (S1 Fig). Then the adjacency matrix was calculated using topological overlap measure (TOM) and corresponding dissimilarity (1-TOM). The latter was used as a distance for gene hierarchical cluster, and then DynamicTree Cut algorithm [46] was used to identify the modules of genes. Modules cluster highly interconnected genes and are named using color labels. The principal component of each module was defined as the module eigengene (ME). The module eigengene represents the expression value of each module and was used to detect biologically relevant modules. The module-trait relationship (module membership, MM) was calculated as the Pearson’s correlation between the module eigengene and the trait of interest.

### Gene Ontology enrichment analysis and identification of hub genes from WGCNA results

Modules significantly associated (*P*-value < 0.05) with IMF deposition were exported for functional analysis in R packages "clusterProfiler" [40] and "AnnotationHub" [41]. The GO enrichment analyses were performed as previously described. The lists of genes entering the significant modules were submitted to functional analysis individually (each module of genes was analyzed separately) and grouped (all the genes entering in the significant modules were analyzed together). Furthermore, the genes in the modules significantly associated with IMF deposition were exported for network and functional analysis in Cytoscape v. 3.7.2 [47] using the function *exportNetworkToCytoscape()* in “WGCNA” package. Using this command, all the genes significantly entering in the IMF-associated modules identified with WGCNA were exported in a unique session in Cytoscape v. 3.7.2. The analysis started building a network of genes using the “GeneMANIA” plugin [48] and then functional analysis was performed using the ClueGO plugin [49]. Then “ClueGO” plugin divided the genes into different functional groups having different *P*-values. Each functional group contained the pathways and biological processes (BPs) clustered together according to term similarities. The statistical method was set at two-sided hypergeometric distribution, and Bonferroni step down *P*-value correction was used. Minimum clustering was set at *P*-value ≤ 0.05 and a minimum k-score at 0.4. The BPs ontology and KEGG and REACTOME pathways were used as databases for the functional analysis. Gene ontology (GO) levels were set from 6 to 8, and a minimum number of 4 genes per cluster was set. Subsequently, “cytoHubba” [50] and “CluePedia” plugins were applied to select and display in the figures the hub genes. The top 10 hub genes were identified using Maximal Clique Centrality (MCC) as a topological analysis method [50].

## Results and discussion

### Description of the studied sample

Table 1 reports the sex, the carcass weights and the SM intramuscular fat % of the studied samples. The analyzed samples were chosen from a population of ILW pigs displaying an average content of IMF of 2.15 ± 1.13% and a carcass weight of 114.50 ± 8.64 kg. As can be noticed from Table 1, the 12 pigs used for the present transcriptome study were characterized by an extreme phenotype for SM IMF content, with the low IMF group composed of 6 samples with 0.51% ≤ IMF ≤ 0.74% and an average IMF content of 0.67 ± 0.09%, and the pigs belonging to the high IMF group displaying an average IMF % of 6.81 ± 1.17%, with 5.87% ≤ IMF ≤ 8.64%. The 12 investigated pigs had an average carcass weight of 120 kg that approaches the weight of typical heavy pigs grown to produce high-quality dry-cured hams, such as Parma and San Daniele. These pigs were slaughtered at about eight months of age, which is quite a different age when compared with the transcriptomics studies performed on porcine muscle. For instance, Muñoz et al. [23] analyzed the transcriptome of muscle gathered from 17 months-old pigs, while other authors used muscle samples collected from animals sacrificed at an average age of 5 to 6 months [25, 51]. Differences in growth performances and slaughter ages characterizing the animals used in the muscle transcriptomics studies could affect the observed results since growth rate and age are associated with distinct differentiation and hypertrophia stages of the adipocytes interspersed in the muscle [9, 28].

### Characterization of *Semimembranosus* muscle transcriptome analysis

**Fig 1:**
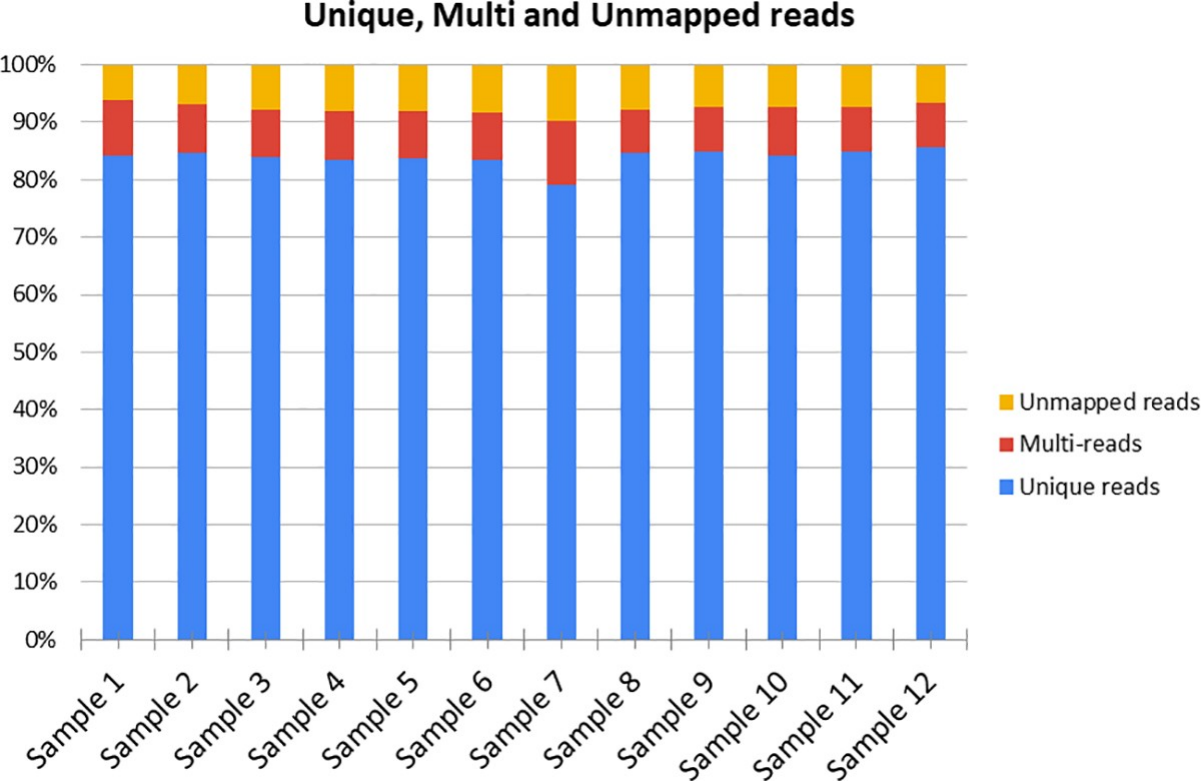
Mapping statistics. For each sample are reported the percentages of the uniquely mapped reads, multi-reads and unmapped reads against the *Sus scrofa* reference genome 11.1.

A total of 3,235,579,132 of paired-end reads were obtained from the SM transcriptome sequencing of the 12 samples. After trimming and filtering, 1,155,025,434 reads remained. Between 79.1% and 85.7% of the reads were uniquely mapped against the *Sus scrofa* reference genome 11.1 across samples (Fig 1 and S2 Table). Considering both the unique and the multi reads, the total % of mapped reads stands between 90 and 95%, in agreement with the data reported in other recent porcine muscle transcriptome studies [23].

### Identification of differentially expressed genes

**Fig 2:**
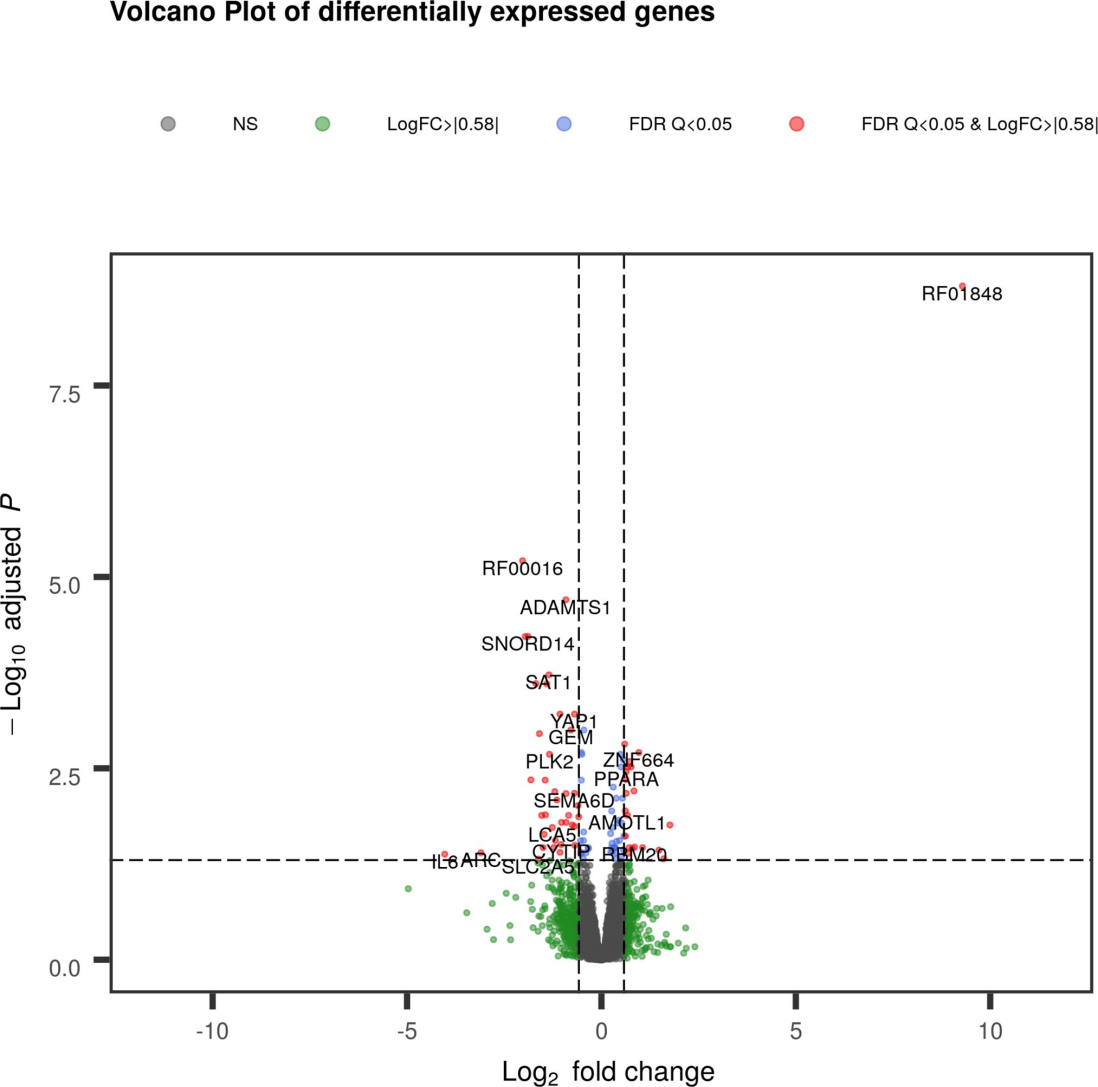
Volcano plot of differentially expressed genes (DEGs) of low Intramuscular Fat (IMF) *vs*. high IMF pigs. Red dots indicate DEGs with q < 0.05 and |Log_2_(Fold Change)| > 0.58; blue dots indicate genes with q < 0.05, green dots represent genes with |Log_2_(Fold Change)| > 0.58, grey dots are non-significant genes. The gene plotted as “SNORD14” refers to ENSSSCG00000039904 RF00016.

**Fig 3:**
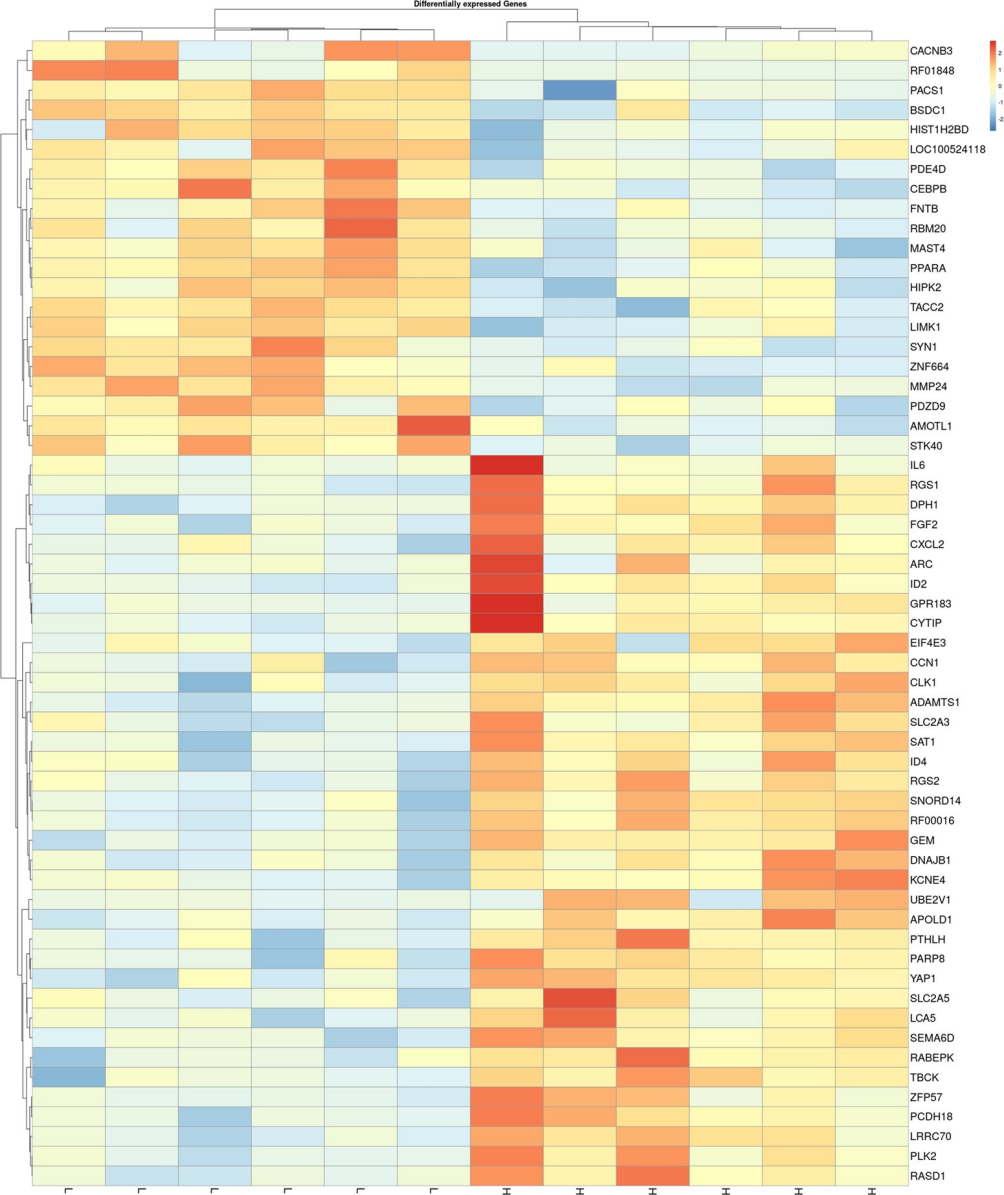
Gene expression unsupervised hierarchical clustering of the 58 differentially expressed genes (DEGs) between low Intramuscular Fat (IMF) *vs*. high IMF pigs. The color scale bar shows the relative gene expression changes normalized by the standard deviation (0 is the mean expression level of a given gene). H and L indicate the high IMF and the low IMF samples, respectively. The gene named “SNORD14” refers to ENSSSCG00000039904 RF00016.

The differential expression analysis showed a total of 58 DEGs with a q value ≤ 0.05 and 31 with a trend towards significance (q ≤ 0.10). The complete list of the identified genes is reported in the S3 Table. Among the 58 DEGs, 37 were up-regulated in the high IMF group with log_2_FC ranging from -4.03 to -0.60, while the remaining 21 DEGs were up-regulated in the low IMF group with log_2_FC ranging from 9.29 to 0.60 (S3 Table). The volcano plot in Fig 2 shows the identified DEGs. As shown in Fig 3, when considering these 58 DEGs the gene expression unsupervised hierarchical clustering properly divides the samples into two groups belonging to different IMF content, thus underlying that their differential regulation may be involved in IMF content and deposition. Table 2 reports a subset of DEGs chosen among the identified 58 for their functional roles possibly associated with IMF deposition.

**Table 2:** List of the main differentially expressed genes (DEGs) associated with Intramuscular Fat (IMF) deposition in porcine *Semimembranosus* muscle.

ENSEMBL Gene ID	Official gene symbol	Average gene expression of the low IMF group	Average gene expression of the high IMF group	Log_2_(FC)^a^	Log_2_(FC) S.E.^b^	Wald statistic	*P*-value	Adjusted *P*-value (q value)
ENSSSCG00000036462	RF01848	4,341.64	7.47	9.29	1.25	7.41	1.31E-13	1.59E-09
ENSSSCG00000032967	*CACNB3*	397.60	131.00	1.61	0.45	3.55	3.82E-04	4.78E-02
ENSSSCG00000034207	*CEBPB*	11,387.99	6,378.77	0.84	0.19	4.32	1.59E-05	6.24E-03
ENSSSCG00000030378	*LIMK1*	9,651.69	5,827.761	0.73	0.16	4.59	4.45E-06	2.58E-03
ENSSSCG00000016929	*PDE4D*	25,069.80	15,664.78	0.68	0.15	4.53	5.99E-06	3.03E-03
ENSSSCG00000000006	*PPARA*	1,883.74	1,212.31	0.64	0.14	4.49	7.25E-06	3.52E-03
ENSSSCG00000028063	*TACC2*	6,476.73	4,230.56	0.61	0.15	4.12	3.84E-05	1.14E-02
ENSSSCG00000036213	*FGF2*	864.10	1,454.32	-0.75	0.19	-3.96	7.59E-05	1.74E-02
ENSSSCG00000006105	*GEM*	652.48	1,126.729	-0.79	0.16	-4.89	9.89E-07	1.00E-03
ENSSSCG00000008645	*ID2*	508.34	955.21	-0.91	0.23	-4.00	6.35E-05	1.61E-02
ENSSSCG00000012026	*ADAMTS1*	3,444.16	6,491.10	-0.91	0.16	-5.85	4.91E-09	1.99E-05
ENSSSCG00000036893	*PTHLH*	57.28	115.94	-1.03	0.26	-3.99	6.50E-05	1.61E-02
ENSSSCG00000011516	*EIF4E3*	1,066.91	2,230.21	-1.06	0.29	-3.63	2.80E-04	3.96E-02
ENSSSCG00000006940	*CCN1*	3,062.23	6,772.00	-1.14	0.27	-4.21	2.57E-05	8.21E-03
ENSSSCG00000004469	*LCA5*	136.10	325.75	-1.26	0.32	-3.93	8.65E-05	1.88E-02
ENSSSCG00000012173	*SAT1*	2,015.31	5,139.85	-1.35	0.25	-5.34	9.39E-08	1.90E-04
ENSSSCG00000024312	*ID4*	488.77	1,333.62	-1.45	0.33	-4.40	1.07E-05	4.52E-03
ENSSSCG00000009517	*GPR183*	76.05	210.52	-1.47	0.38	-3.86	1.12E-04	2.30E-02
ENSSSCG00000022925	*SLC2A3*	1,356.71	3,937.21	-1.54	0.38	-4.07	4.75E-05	1.30E-02
ENSSSCG00000013784	*DNAJB1*	5,418.77	16,383.87	-1.60	0.33	-4.86	1.19E-06	1.11E-03
ENSSSCG00000039651	*SLC2A5*	203.42	625.64	-1.62	0.46	-3.55	3.92E-04	4.85E-02
ENSSSCG00000037241	*RGS2*	789.87	2,539.31	-1.68	0.32	-5.26	1.42E-07	2.47E-04
ENSSSCG00000039904	RF00016	115.66	427.28	-1.89	0.34	-5.58	2.47E-08	6.01E-05
ENSSSCG00000031725	RF00016	130.89	509.37	-1.96	0.35	-5.58	2.42E-08	6.01E-05
ENSSSCG00000035039	RF00016	86.14	350.98	-2.03	0.33	-6.11	1.01E-09	6.15E-06
ENSSSCG00000020970	*IL6*	62.87	1,022.05	-4.03	1.12	-3.60	3.20E-04	4.18E-02

^a^ log_2_(Fold Change) of the gene expression levels in the low IMF group *vs*. the high IMF group.

^b^ Standard error (S.E.) of the log_2_(Fold Change)

The two most significant DEGs were ENSSSCG00000036462 RF01848 (log_2_FC = 9.29 and adjusted *P*-value = 1.59E-09) and ENSSSCG00000035039 RF00016 (log_2_FC = -2.03 and adjusted *P*-value = 6.15E-06). Both these genes are predicted non-coding small nucleolar RNAs (snoRNAs), a class of regulatory RNAs consisting of 60–300 nucleotides responsible for the post-transcriptional modification, maturation and stabilization of ribosomal RNAs [52, 53]. The snoRNA RF01848 (ENSSSCG00000036462) is predicted to have an ACEA_U3 skeleton structure, whereas ENSSSCG00000035039 RF00016 was predicted to have a SNORD14 skeleton. At present, ENSSSCG00000035039 RF00016 has been retired from the latest release of *Sus scrofa* genome annotation in Ensembl database, but together with ENSSSCG00000035039, also two other snoRNAs with a SNORD14 skeleton structure were more expressed in the high IMF group: the ENSSSCG00000039904 RF00016 (log_2_FC = -1.89; adjusted *P*-value = 6.01E-05) and ENSSSCG00000031725 RF00016 (log_2_FC = -1.96; adjusted *P*-value = 6.01E-05). Recently, equine adipose-derived mesenchymal stromal cells were proven to release extracellular vesicles mainly enclosing snoRNAs [54], suggesting that this type of regulatory non-coding RNAs may play an active part in cell differentiation and vesicle-mediated cross-talk between cells. Intriguingly, some SNORDs were also involved in intracellular cholesterol trafficking and its mobilization from the plasma membrane to the endoplasmic reticulum [53, 55]. Intracellular cholesterol homeostasis is essential in adipocytes, which functions as a primary depot of unesterified cholesterol in the body [56]. Taken together, these results reported in the literature would suggest a role in adipocyte proliferation and cholesterol trafficking of some SNORDs. Our results agree with the results found in the literature for some SNORDs and may indicate that the SNORDs found DE in the present research could be involved in some of the molecular networks related to IMF deposition. However, further evidence is needed to elucidate the roles of SNORDs in muscle and prove their possible involvement in the proliferation and metabolism of the muscle-interspersed adipocytes. Interestingly, some genes found DE in the present study are already known in the scientific literature for their roles in adipogenesis and lipid metabolism. Among them, *Peroxisome Proliferator Activated Receptor Alpha* (*PPARA*) was already reported in the literature to regulate the expression of many genes critical for lipid and lipoprotein metabolism and was found to be highly expressed in tissues that have a high level of fatty acid catabolism [57]. Indeed, the expression of *PPARA* promotes fatty acid β-oxidation mediating the activation of genes intervening in lipids catabolism [58], with beneficial effects on liver steatosis, and lowering effects on plasma triglycerides and small dense low-density lipoproteins [59]. These anti-adipogenic effects noticed for the human *PPARA* gene agree with the negative correlations identified in pigs between loin IMF content and *PPARA* mRNA level [60, 61]. Consistently, we identified higher expression of *PPARA* in the pigs belonging to the low IMF group (log_2_FC = 0.64; adjusted *P*-value = 3.52E-03), supporting the anti-adipogenic role exerted by this transcription factor on IMF deposition. Similarly, also *CCAAT Enhancer Binding Protein Beta* (*CEBPB*) was more transcribed in low IMF pigs (log_2_FC = 0.84; adjusted *P*-value = 6.24E-03). Most of the scientific literature indicates *CEBPB* as an early marker of adipogenesis controlling fatty acid metabolism and inflammation in different tissues [62, 63]. The highest expression noticed in low IMF pigs for this gene may be a suggestive marker of undifferentiated pre-adipocytes in an early stage of differentiation, when the commitment of *CEBPB* seems to be crucial for triggering the following stage of maturation and fat droplet formation [64–66]. On the other hand, the *CEBPB* down-regulation in high IMF group may suggest that in these individuals the muscle interspersed adipocytes have undergone a more advanced differentiation stage, with the formation of lipid droplets. The latter stage does not require the expression of the *CEBPB* gene, which was found down-regulated in mature adipocytes [64–66]. In the two groups of samples, the mRNA amounts of the gene *Fibroblast Growth Factor 2* (*FGF2*) showed the opposite trend compared to *CEBPB* levels. *FGF2* is synthesized and secreted by adipocytes [67], and depending on its concentration, *FGF2* can function as either a positive or negative factor of in vitro adipogenesis through the regulation of the ERK signaling pathway [68]. FGF family members are involved in a variety of biological processes including embryonic development, cell growth, and morphogenesis [69]. In particular, polymorphisms in the *FGF2* gene were already associated with bovine milk fat yield and percentage [70, 71], with fat-related traits in pigs [72] and with human body fat mass [73]. Thus, the increased expression of *FGF2* noticed in pigs with high IMF (log_2_FC = -0.75; adjusted *P*-value = 1.74E-02) may be the result of the increased activation of this gene and secretion of the relative growth factor by the differentiated and hypertrophic adipocytes interspersed in the SM of the high IMF animals. Recently, FGF2 was also studied for its effects on primary cilia, a solitary non-motile cilium that projects from the apical surface of cells to the internal lumen of the tissues and acts as a sensory antenna transducing a multitude of chemical and physical stimuli [74]. Kunova Busakova et al. [75] found that mesenchymal cells treated with FGF2 showed an elongation of primary cilia and activation of phosphatidylinositol-3-kinase (PI3K)/AKT, mammalian target of rapamycin (mTOR) signaling and ERK MAP kinase signaling. Interestingly, in agreement with the evidence concerning FGF2-related effects reported by Kunova Busakova et al. [75], among the DEGs we found several genes participating in primary cilia morphogenesis, and in the PI3K/AKT and ERK MAP kinase signaling. Indeed, compared with low IMF group, high IMF pigs showed also higher expression of *Lebercilin 5* (*LCA5*, log_2_FC = -1.26; adjusted *P*-value = 1.88E-02), a gene coding for a protein intervening in the primary cilia morphology [76], and increased expressions of *Cellular Communication Network Factor 1* (*CCN1*, *alias CYR61*, log_2_FC = -1.14; adjusted *P*-value = 8.21E-03), *G Protein-Coupled Receptor 183* (*GPR183*, log_2_FC = -1.47; adjusted *P*-value = 2.30E-02) and *Interleukin 6* (*IL6*, log_2_FC = -4.03; adjusted *P*-value = 4.18E-02). All the three genes *CCN1*, *GPR183* and *IL6* activate or are activated by the ERK MAP kinase signaling pathway [77–79]. The differential expression noticed for *IL6* in high IMF pigs may also be related to the secretion from white adipose tissue of IL6 [80]. IL6 is a proinflammatory cytokine produced by activated immune cells and stromal cells, including T cells, monocytes/macrophages, endothelial cells, fibroblasts, and hepatocytes [81]. The proteins encoded by this gene have many functions in the regulation of the immune system and metabolism, and play also a role in the body's defense against infection, in many regenerative processes, and in the regulation of body weight [reviewed in 81]. Interestingly, in humans, omental *IL6* mRNA expression correlated negatively with insulin sensitivity and positively with steatosis [82], supporting a role for this gene in energy metabolism and obesity. Other genes found DE were related to the control of cell differentiation and stem cell totipotency, such as *Inhibitor of DNA binding 4* (*ID4*) [83] and *Inhibitor of DNA binding 2* (*ID2*) [84], and to transcription regulation, such as *Eukaryotic Translation Initiation Factor 4E Family Member 3* (*EIF4E3*). The latter was found over-expressed in high IMF individuals compared with the low IMF group (log_2_FC = -1.06; adjusted *P*-value = 3.96E-02), suggesting its possible role in IMF deposition. Our result seems to agree with the role shown by EIF4E in cell proliferation and adipocyte differentiation reported by Nogueira et al. [85]. Indeed, *EIF4E3* is a translational initiation factor [86], but this gene is also required in the AKT/mTORC1/eIF4E axis for adipocyte differentiation [85] since the mammalian target of rapamycin complex 1 (mTORC1) enables EIF4E to interact with EIF4G and to initiate the mRNA translation during adipocyte differentiation [87]. Another gene related to the same gene family (namely *Eukaryotic Translation Initiation Factor 4E Binding Protein 1*, *EIF4EBP1*) was also found among the DEGs associated with porcine backfat thickness [88], supporting the evidence that *EIF4E* gene family may be involved in the processes associated with adipogenesis. mTORC1 signaling was also found to control polyamine synthesis [89]. Interestingly, high IMF pigs showed also a higher expression of *Spermidine/spermine N1-acetyltransferase* (*SAT1;* log_2_FC = -1.35; adjusted *P*-value = 1.90E-04), an enzyme acting in the homeostasis of polyamines [90], that are molecules essential for cell growth [91]. The concurrent increased expression of both *SAT1* and *FGF2* genes noticed in the present research in high IMF pigs is in agreement with the evidence reported in the literature that FGF2 concentrations positively regulate polyamine synthesis in arterial smooth muscle cells [92].

Similarly, high IMF group presented also a higher gene expression for *ADAM Metallopeptidase With Thrombospondin Type 1 Motif 1* (*ADAMTS1*, log_2_FC = -0.91; adjusted *P*-value = 1.99E-05). These shreds of evidence are consistent with the scientific literature concerning this gene. ADAMTS1 is mainly recognized to be involved in extracellular matrix degradation [93], but evidence suggests also its participation in adipogenesis. *ADAMTS1* gene had indeed its expression up-regulated during adipogenesis of human mesenchymal stem cells in the study by Hung et al. [94], and the targeted inactivation of the murine *ADAMTS1* gene resulted in morphological defects in the adipose tissue [95].

**Fig 4:**
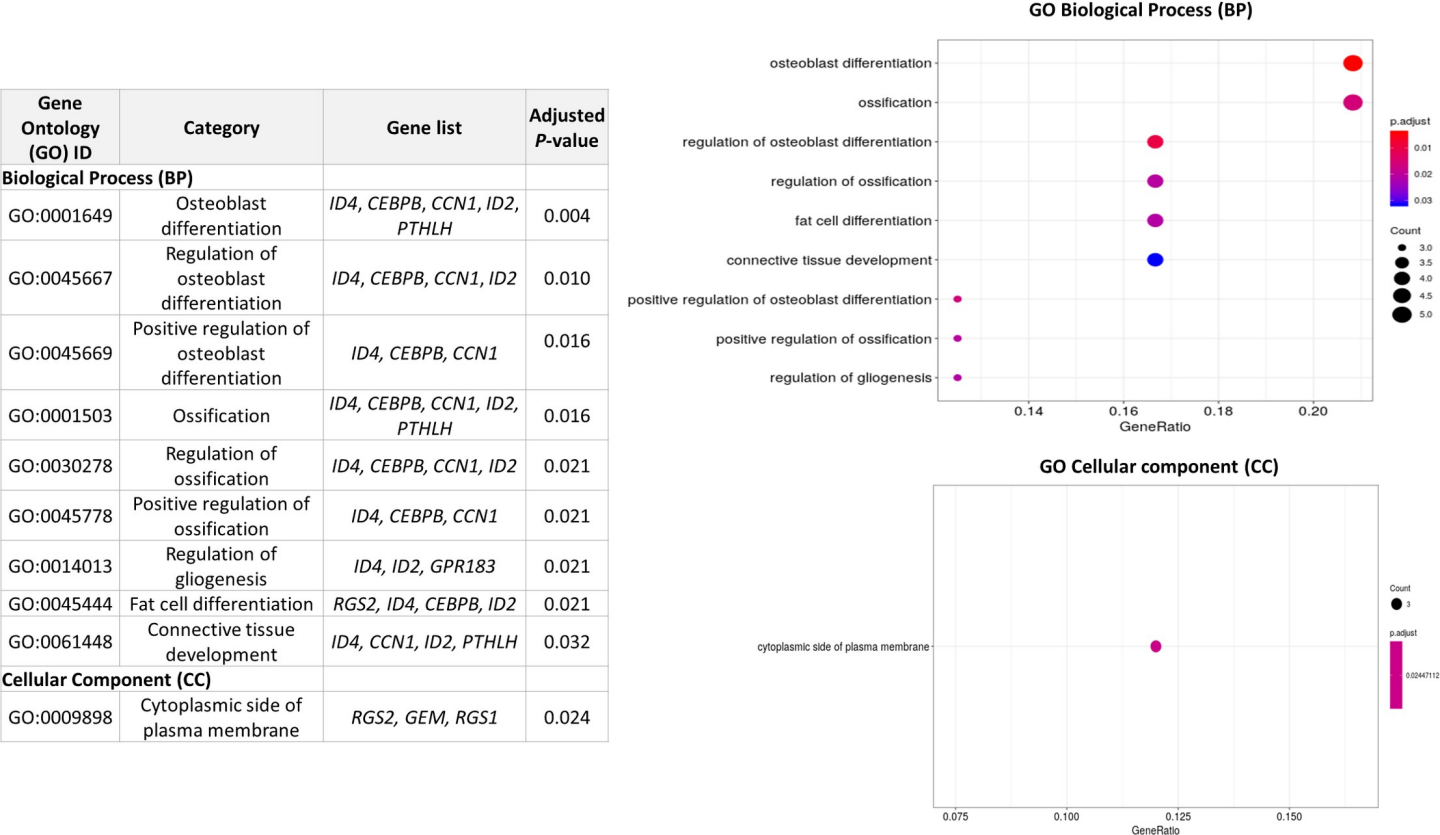
Results of the Gene Ontology (GO) analysis of the 58 differentially expressed genes (DEGs) associated with Intramuscular fat (IMF) deposition. The color and size of the dots show the significance and the ratio between the number of DEGs found in the present study belonging to the functional categories and the total number of genes in the functional categories.

The GO analysis revealed several BPs to be enriched, among which "GO:0014013" corresponding to "Regulation of Gliogenesis" (Adjusted *P-*value = 0.021), "GO:0045444" corresponding to "fat cell differentiation" (Adjusted *P-*value = 0.021) and "GO:0061448" (Adjusted *P-*value = 0.032) which implies a connective tissue development (Fig 4). As can be seen in Fig 4, the genes included in the significant functional categories are almost the same in all the GO terms and are mainly related to the differentiation of precursors to different types of cells (such as fat cells, osteoblasts, glial cells, and fibroblasts). This result is therefore in agreement with the roles previously described for several DEGs, which were found in the literature to be mainly associated with the regulation of cell differentiation and proliferation.

### RT-qPCR validation of differential expression analysis

**Fig 5:**
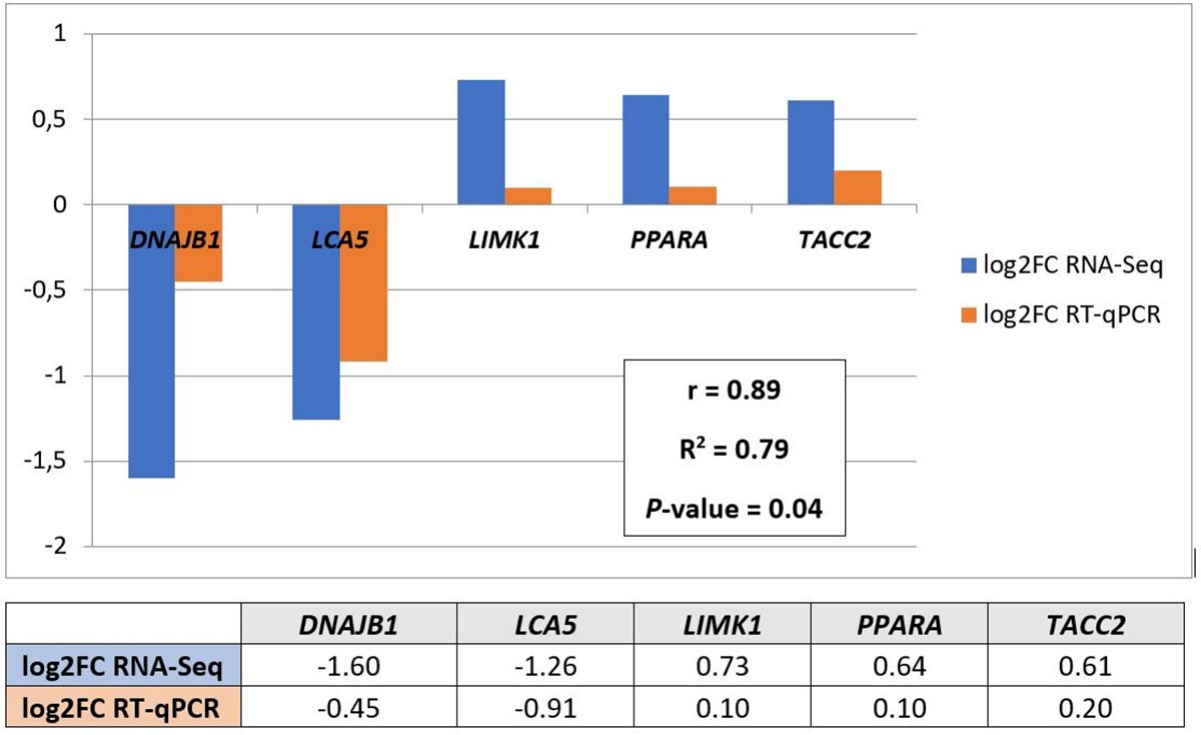
RT-qPCR validation of five genes found differentially expressed by RNA-Seq analysis. The table reports the Log_2_ Fold Change of the gene expressions between low IMF and high IMF groups for the RNA-Seq and RT-qPCR. The same values are also graphically presented with the results of the correlation analysis (with the r correlation coefficient, the R^2^ coefficient of determination, and the *P-*value).

In order to validate the RNA-seq experiment, RT-qPCR was used to assess the expression of five genes (two downregulated and five upregulated in the low IMF group): *DnaJ Heat Shock Protein Family (Hsp40) Member B1* (*DNAJB1*), *LCA5*, *LIM Domain Kinase 1* (*LIMK1*), *PPARA* and *Transforming Acidic Coiled-Coil Containing Protein 2* (*TACC2*). For the selected genes, the value of the Log_2_FC from the RNA-seq analysis was compared to the Log_2_FC obtained with RT-qPCR. The genes selected for the validation showed similar expression patterns between the RNA-seq and the RT-qPCR analyses (Fig 5). Indeed, the Log_2_FC values obtained by RT-qPCR were significantly correlated with those obtained from RNA-seq (r = 0.89, *P*-value = 0.04) and displayed a high coefficient of determination (R^2^ = 0.79; Fig 5).

### Identification of weighted gene correlation networks associated with intramuscular fat content

To detect possible gene correlation networks associated with IMF deposition and add information to the list of DEGs, the package “WGCNA” was used [46]. IMF content showed to be significantly correlated with the genes clustered in four modules: grey60 (module eigenvalue = 0.77; *P*-value = 0.003), darkturquoise (module eigenvalue = 0.65; *P*-value = 0.022), skyblue1 (module eigenvalue = 0.65; *P*-value = 0.022), and lavenderblush3 (module eigenvalue = -0.62; *P*-value = 0.030; Table 1 in S1 File). Firstly, the genes grouped in the significant modules were compared with the list of DEGs obtained using the DESeq2 package. Fifty-four genes out of the 58 identified as DE were found to have an expression level significantly correlated with IMF content, suggesting that there was an overall agreement between the two methods.

The GO analysis of the genes significantly entering in the four modules associated with the IMF content showed only two significant MFs enriched in the skyblue1 module, namely “GO:0045309 Protein phosphorylated amino acid binding” (comprising the genes *CRK Like Proto-Oncogene*, *Adaptor Protein- CRKL*, *Growth Factor Receptor Bound Protein 2*- *GRB2*, *LEO1 Homolog*, *Paf1/RNA Polymerase II Complex Component*- *LEO1*, *NCK Adaptor Protein 2- NCK2*, *RAS P21 Protein Activator 1- RASA1*; Adjusted *P-*value = 0.010) and “GO:0001784 Phosphotyrosine residue binding” (with *CRKL*, *GRB2*, *NCK2*, and *RASA1*; Adjusted *P*-value = 0.040).

**Fig 6:**
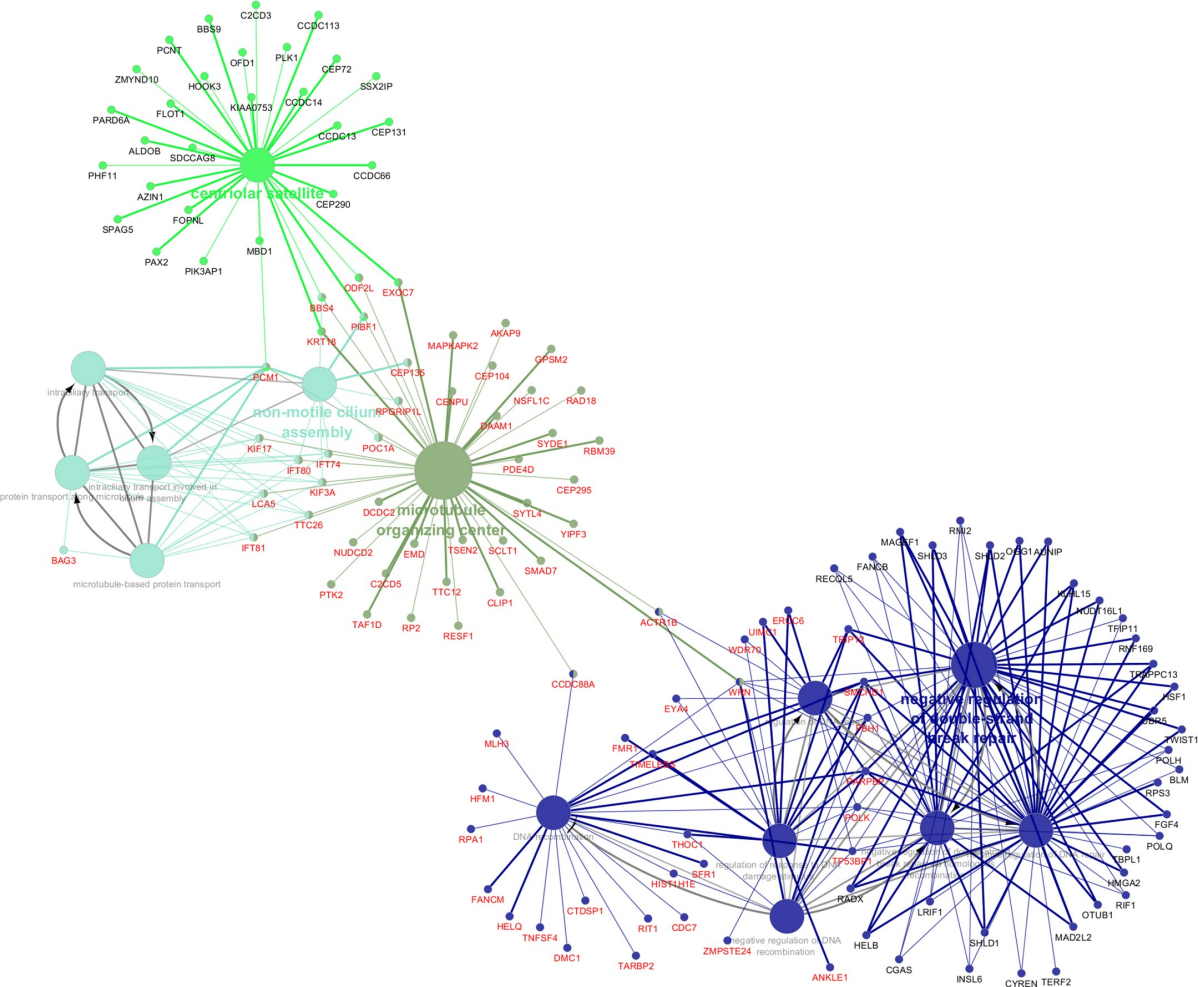
Results of the Cytoscape functional analysis of the genes found in the four significant modules associated with Intramuscular fat (IMF) deposition in the *Semimembranosus* muscle. Different functional categories are represented in different colors. The genes with central roles in these categories are also plotted.

The genes in the four significant modules associated with porcine IMF content were further investigated in Cytoscape. The complete lists of gene module memberships with the *P*-values and the correlation coefficients are reported in Tables 2 and 3 in S1 File. The overall significances of the genes for the IMF content and the relative *P*-values are reported in Table 4 in S1 File. Fig 6 shows the significant GO BPs and CCs identified for the list of genes belonging to grey60, darkturquoise, skyblue1, and lavenderblush3 modules. The results of the functional categories related to the genes found with the weighted gene correlation analysis highlighted two macro-categories of genes: a first cluster of genes closely linked to the regulation of cell differentiation, DNA transcription in the cell nucleus, alternative splicing of transcripts, maturation and translation of mRNA, and a second group of genes linked to the cellular structure (centriolar satellite, microtubule organizing center and non-motile cilium assembly). The complete list of the GO terms, the genes found to be associated and the *P*-values are reported in S4 Table.

Of particular interest is the latter functional category, which contains the DE gene *LCA5*, and several genes that code for proteins that fall within the organization of primary cilia, such as the various intraflagellar transport proteins (i.e. *IFT81*, *IFT80*, *IFT74*) and centrosomal proteins (*CEP135*). As previously discussed, the scientific literature is recently investigating with increasing interest in the role played by primary cilia in cellular energy metabolism. While mature adipocytes are not thought to be ciliated, a transient primary cilium has been described during the differentiation of preadipocytes [96], with ciliary proteins expressed during adipogenesis [97]. These specialized cellular organelles are formed during interphase of the cell cycle from an ancestral basal body or elder centriole of the centrosome, to which primary cilia remain closely connected. The prominent roles of these organelles in cell differentiation and energy metabolism are only recently beginning to be understood. Indeed, the primary cilia play critical roles associated with the epithelium–mesenchyme interaction in various tissues, and several studies evidenced that the primary cilia surface comprises receptors for many growth factors and chemical stimuli which permit the cross-talk between adjacent tissues and the regulation of the development and functional differentiation [98]. Primary cilia are also found to express on their surface receptors associated with the regulation of intracellular energy balance. Indeed, rat tracheal ciliated cells presented glucose transporters (GLUT family) on their surface [99]. In the present study, we found *Solute Carrier Family 2 Member 5* (*SLC2A5*, *alias GLUT5*) up-regulated in the high IMF group (Table 2). This gene encodes a fructose transporter responsible for fructose uptake in cells and was proved to be essential in the adipocyte differentiation process [100, 101]. These results are therefore in agreement with our findings, and it could be hypothesized that *SLC2A5* expression in adipocytes may be dependent also on ciliation events. Anyway, the literature concerning *SLC2A5* and its possible relation with primary cilia is still scant, and this interpretation would need further dedicated studies to be proved. Along with *SLC2A5* gene, also the *Solute Carrier Family 2 Member 3* (*SLC2A3*, *alias GLUT3*) was found up-regulated in the high IMF group (Table 2). This solute carrier mediates the uptake of glucose and various other monosaccharides (except fructose) across the cell membrane. *SLC2A3* was mainly found expressed in nerve cells [102], but its gene and protein expression were also detected within the human myocytes and in particular appear much present in the nerves within the muscle sections [103]. Its expression in muscle seems to be associated with regenerative muscle fibers [104], but our results along with those reported in beef cattle [105] may also suggest for this gene a possible involvement in the molecular cascade associated with divergent IMF deposition in livestock species. Among the receptors located to primary cilia are also G protein–coupled receptors (GPCRs), such as neuropeptide Y (NPY) receptors, commonly associated with the regulation of energy balance and feed intake [106]. Intriguingly, in the present research, we found several DEGs involved in G-protein signaling, such as the already cited *GPR183*, suggesting also a possible relationship exists between primary cilia and the intracellular cascade following chemical stimuli. This hypothesis is also supported by the fact that the GPR183 receptor binds to oxysterols [107], bioactive lipids derived from cholesterol that are mediators of obesity and inflammation [108]. Abnormal primary cilia morphology was associated with obesity and insulin resistance in humans [97, 109], proving the primary role played by this still poorly known organelle in cell energy metabolism. Such findings are even more interesting when taken into context with the results reported in the literature concerning the strong linking relating cilia morphogenesis and transcriptional changes in the cell nucleus [110] and the role of FGF signaling in the regulation of cilia morphogenesis [111]. In agreement with the reported literature, we found higher *FGF2* gene expression in high IMF pigs, and similar expression patterns were observed also for genes related to intracellular signaling pathways and transcription regulation. The cluster of genes involved in transcription regulation is connected with the microtubule gene set through the genes *Actin Related Protein 1B* (*ACTR1B*, *alias ARP1B*) and *WRN RecQ Like Helicase* (*WRN*; Fig 6). *ACTR1B* significantly entered in grey60 and darkturquoise modules (Tables 2 and 3 in S1 File) and displayed a negative correlation with the IMF amount (Table 4 in S1 File). *ACTR1B* belongs to the gene family of Actin Related Proteins (ARPs). ARPs function largely or entirely in the nucleus, and participate together with actin in chromatin remodeling, transcription and nuclear assembly [112]. ARPs have crucial roles in actin polymerization, which in turn was found to control primary cilia morphogenesis and the related intracellular signaling pathways [113]. The disruption of actin polymerization, or the knockdown of the involved genes, resulted in an increase in ciliation frequency, axoneme length, and intracellular cilia-related signaling in cultured cells [113]. These findings provide useful insight to guide the interpretation of the expression patterns we found for *ACTR1B* and cilia-related genes. Indeed, the concomitant down-regulation of *ACTR1B* and up-regulation of cilia morphology related genes (*LCA5*, intraflagellar and centrosomal genes) noticed in the present study may suggest that ciliation events and disruption of actin polymerization may have taken place in high IMF pigs.

**Fig 7:**
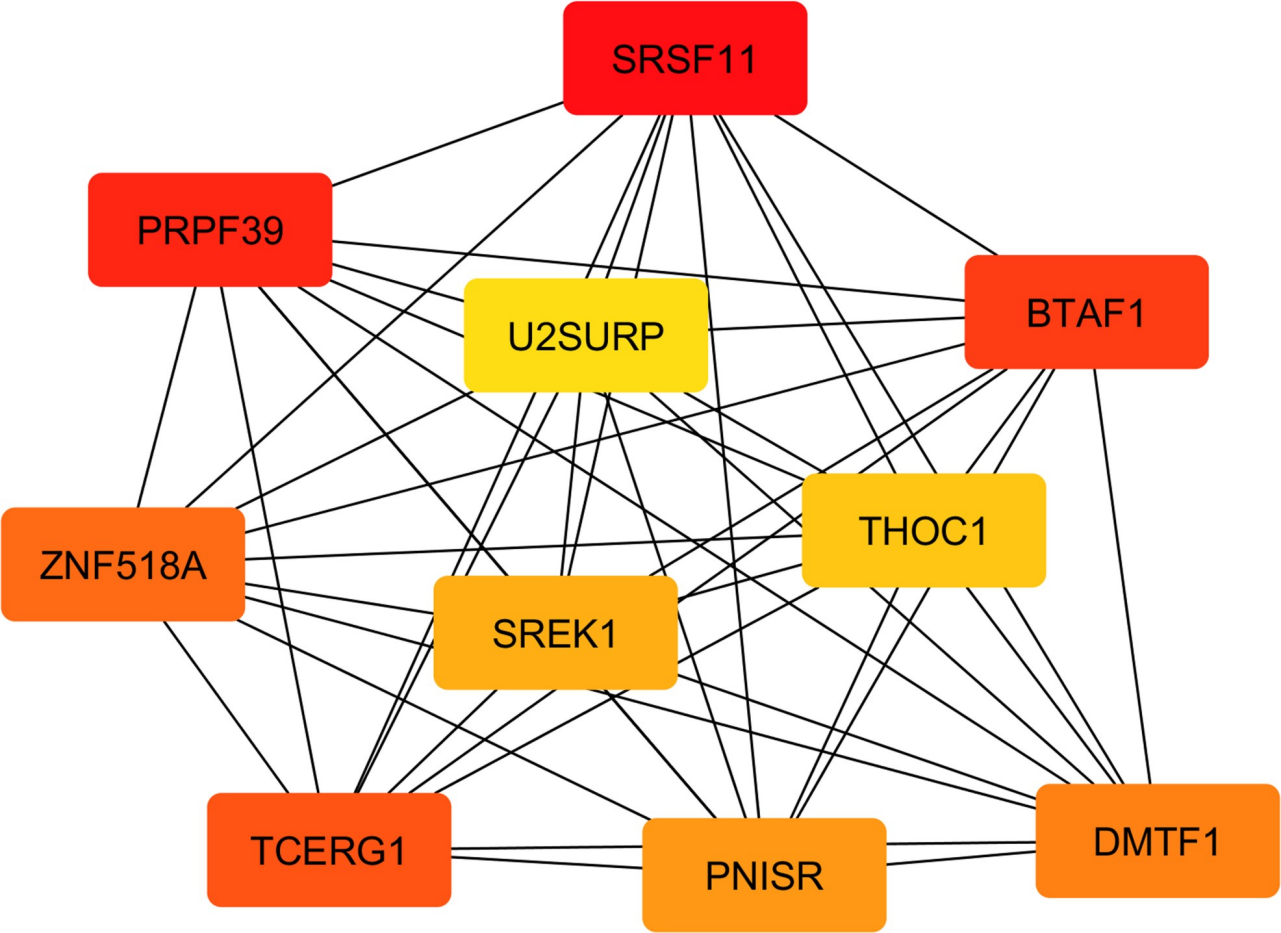
The top 10 hub genes in the network of the co-expressed genes found in the four significant modules. The intensity of the color shows the ranking position: the dark red genes have the most significant Maximal Clique Centrality (MCC) values and thus are hub genes of greater importance in the network; the light-yellow ones have lower MCC values and thus are hub genes of lower importance in the network.

The identification of hub genes with the Cytoscape plugin “cytoHubba” showed a rank of 10 genes, graphically presented in Fig 7.

Almost all the ten identified hub genes encode for proteins falling into the spliceosome, one of the most complex of the cell molecular machines comprising the coordinated interaction of more than 150 proteins involved in RNA splicing [114]. The three genes showing the highest values of MCC, and thus being reported in darker red in Fig 7, were *B-TFIID TATA-Box Binding Protein Associated Factor 1* (*BTAF1*), *Splicing factor serine-arginine rich protein* (*SFRS11*), and *Pre-mRNA Processing Factor 39* (*PRPF39*). BTAF1 is a TATA-box binding protein (TBP) associated factor that regulates TBP thus controlling the dynamic cycling of TBP on and off of DNA and its transcription into RNA [115]. Of great interest are the results recently published by Hardivillé et al. [116], which showed how changes in the interaction between BTAF1 and TBP lead to gross alterations in lipid storage, suggesting that this gene may have a consistent role also on the regulation of the transcriptomic cascade associated with differential IMF deposition. Anyway, the latter hypothesis would need further specific studies to be proved. The *PRPF39* gene (Fig 7) is still poorly investigated, but other members belonging to the same family of pre-mRNA processing factors (PRPFs) have been studied due to their involvement in retinal diseases [117]. The PRPFs are components of the U4/U6.U5 tri-small nuclear ribonucleoprotein subunit of the spliceosome, catalyzing pre-mRNA splicing. Interestingly, transcripts encoding components of retinal photoreceptor primary cilia were found to be affected by a specific splicing program, and mutations in the sequence of another PRPF family member (namely *PRPF31*) were found to affect ciliogenesis [117]. Although these results concern another gene of the *PRPF* family, it could be hypothesized that *PRPF39* may also have some role in the molecular processes associated with primary cilia. This hypothesis, however, would require further evidence to be proven. Another splicing factor identified among the hub genes in Fig 7 is *Serine And Arginine Rich Splicing Factor 11* (*SFRS11*, *alias P54*). The mRNA level of *SRSF11* positively correlated with the genes in the darkturquoise module (Tables 2 and 3 in S1 File), where also the already discussed *ACTR1B* was clustered. *SRSF11* is a pre-mRNA splicing factor and our results seem to indicate that this gene may have an important role during the splicing events related to adipogenesis in muscle. This hypothesis seems to agree with the findings reported by Lin et al. [118] for *SRSF6*, another member of SRSF gene family, which is required to drive the transcriptional changes related to brown adipocyte differentiation [118]. Thus, it could be hypothesized also for *SRSF11* a possible role in the cell cycle events during the preadipocyte differentiation. Anyway at present the scientific literature describes the roles of this gene in the cell cycle of carcinogenic cell lines [119]. To our knowledge, no specific literature reporting its possible roles in adipocyte differentiation exists. This hypothesis is also supported by the fact that several other genes involved in cell-cycle were already reported to play a central role also in coordinating the transition between cell proliferation and terminal differentiation of preadipocytes [120]. Interestingly, *SFRS11* was not the only member of the Serine And Arginine Rich Splicing Factor family identified among the hub genes. Together with *SFRS11*, also the gene encoding for *PNN Interacting Serine And Arginine Rich Protein* (*PNISR; alias SFRS18*) and *Splicing Regulatory Glutamic Acid And Lysine Rich Protein 1* (*SREK1*, *alias SFRS12*) were found to have a central role in the gene expression network identified in the present research (Fig 7). In detail, PNISR is a serine-arginine rich splicing factor participating in the pre-mRNA splicing machinery [121, 122]. Interestingly, the scientific literature on *PNISR* throws light also upon its possible role of paramount importance in porcine muscle adipogenesis. Indeed, Wang et al. found that differential expression of *PNISR* gene in muscle is correlated with IMF content in pigs and hypothesized that this evidence may be due to a possible implication of the *PNISR* gene in the pre-mRNA splicing of key genes regulating IMF deposition [123]. Additionally, members of the serine-arginine rich splicing factor family were also found involved in ciliogenesis [124], supporting once again the hypothesis of a relationship linking some of the hub genes clustered in the DNA transcription regulation with primary cilia organelle development and morphogenesis.

Two other hub genes identified by “cytoHubba” were *U2 SnRNP Associated SURP Domain Containing* (*U2SURP*, *alias SR140*) and *Zinc Finger Protein 518A* (*ZNF518A*; Fig 7). The gene expression of *U2SURP* was in particular found to be significantly correlated with all the four modules associated with the IMF content (Tables 2 and 3 in S1 File). In agreement with the previously described results, also this gene codes for a protein directly involved in the spliceosome machinery [114], but at present, its function remains mostly unknown [125]. A few researches concerning U2SURP protein associate its activation to variations in intracellular Ca^2+^, which in turn impacted also on cellular growth and proliferation [126]. The involvement of calcium channels as co-regulators of the cell proliferation and transcriptional processes was proved by several studies, where specific patterns of cytoplasmic Ca^2+^ signals were found to control cell proliferation and execution of transcriptional programs [127, 128], while dysfunctional intracellular Ca^2+^ channels may affect cellular transformation and tissue remodeling in various pathologies [129]. Interestingly, intracellular Ca^2+^ signaling was proved to be activated by FGF2 in satellite cells, activation that was found to be essential in the differentiation process [130]. This latter evidence is far more of interest considering that in the present research we found DE genes involved in calcium-channel complex, such as *Calcium Voltage-Gated Channel Auxiliary Subunit Beta 3* (*CACNB3*) and *Phosphodiesterase 4D* (*PDE4D*; Table 2). Hence, based on the evidence linking Ca^2+^ channels and transcription regulation [129, 131], it could be outlined a possible relationship between the identified hub genes involved in the spliceosome machinery and the genes encoding for calcium-channel complex found DE in the present study.

**Fig 8:**
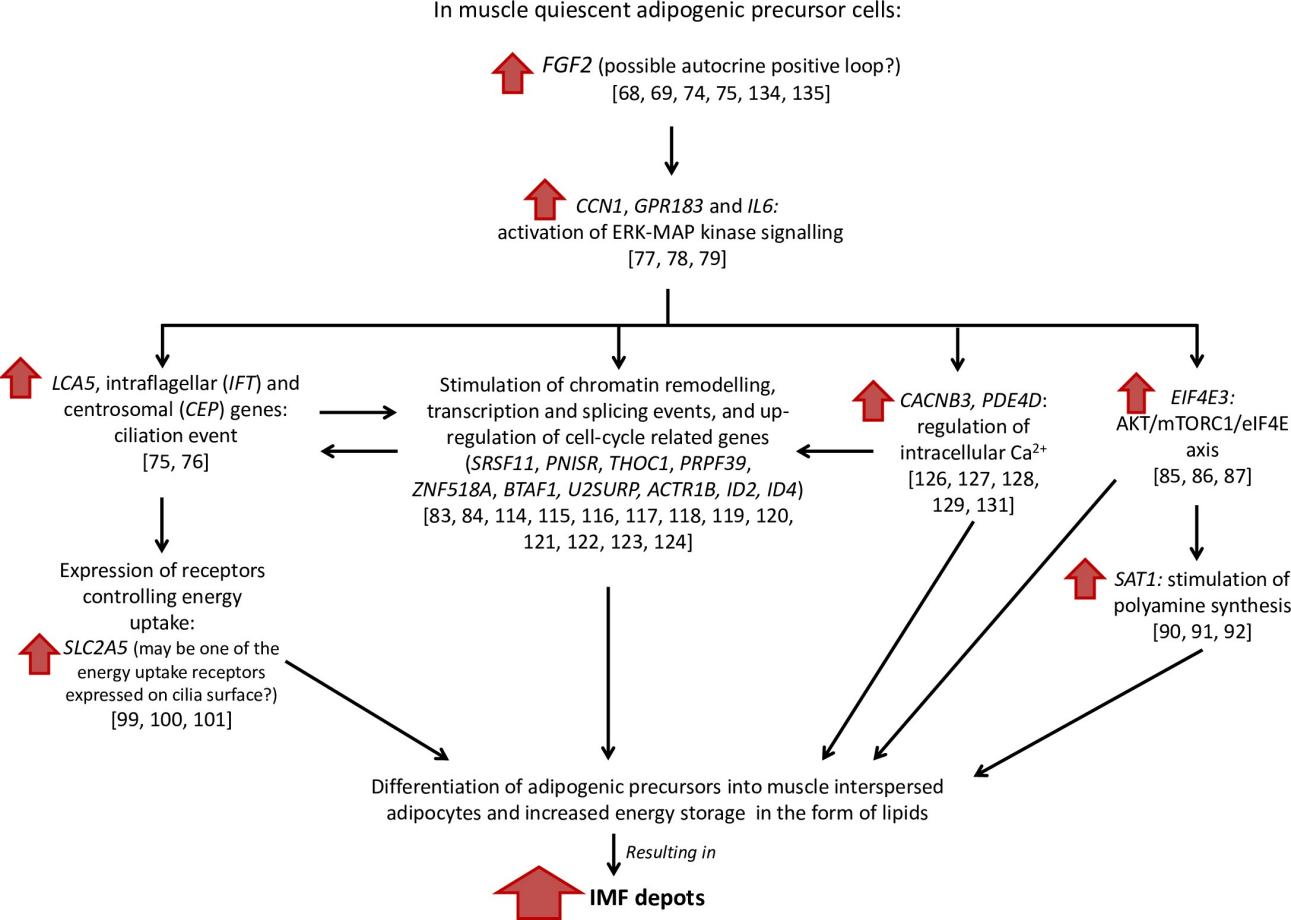
A hypothetical molecular cascade of events involved in Intramuscular fat (IMF) deposition based on the obtained results and comparison with the scientific literature. Between brackets are reported the references endorsing each step in the hypothetical cascade of molecular events.

Another hub gene is *ZNF518A*, which belongs to zinc finger proteins (ZFPs), one of the largest classes of transcription factors in eukaryotic genomes. Despite the exact role of *ZNF518A* is still unknown, many of the ZFPs were found to be involved in the regulation of normal growth and development of cells and tissues through diverse signal transduction pathways [132]. Furthermore, recent studies have found that an increasing number of ZFPs could function also as key transcriptional regulators involved in adipogenesis [132, 133], possibly indicating that also *ZNF518A* may play an important role in the differentiation of muscle interspersed adipogenic precursors. The pluripotency of stem cells was also shown to strongly depend on the THO complex, which is a nuclear protein complex functioning as an interface between mRNA transcription and export [60]. THO complex comprises also the protein encoded by the gene *THO Complex 1* (*THOC1*, *alias P84*), that is one of the hub genes identified in the IMF-related gene network (Fig 7) and with a significant weight in the grey60 gene module (Tables 2 and 3 in S1 File). Therefore, the present research strongly supports that the found hub genes may have a central role also in coordinating the transition between cell proliferation and terminal differentiation of preadipocytes interspersed in muscle. However, based on the knowledge reported in the literature, we believe that upstream of these transcriptional changes there could be an autocrine positive loop driven by FGF2. A possible hypothesis of the molecular cascade of events involved in IMF deposition is outlined in Fig 8.

In particular, considering the effects played by FGF2 in the regulation of cell proliferation [134], primary cilia morphology [75], adipocyte differentiation [135] and polyamine synthesis [92], we suggest that the observed gene networks related to IMF deposition may be driven by *FGF2*. In the literature, a study carried out on human multipotent adipose-derived stem cells (hMADS) proved that FGF2 is critical to maintain the differentiation capacity of these cells and to stimulate their growth also at the single-cell level [135]. This type of regulation of the cell differentiation seems to be carried out through an autocrine loop since FGF2 was proved to be exported to hMADS cell surface to bind with its receptors without being released outside of the cell [135]. Taking into account the scientific literature and the observed increased expression of the primary cilia-related genes, in the SM of high IMF pigs, it is possible to hypothesize the occurrence of ciliation events affecting the adipocyte differentiation process and the stimulation of energy uptake and storage in the form of adipocyte intracellular lipids. This hypothesis agrees with the recent scientific literature indicating that primary cilia are necessary for the differentiation of human adipose progenitors in muscle [136, 137]. Adipogenesis would also be sustained by an increase in polyamine synthesis through the activation of the *SAT1* gene by the *FGF2* signal, in agreement with the results reported in Endean et al. [83]. This complex molecular cascade would anyway need further dedicated studies to confirm the outlined hypothesis.

## Conclusions

On the whole, the genes identified in this study associated with IMF in pigs were mainly involved in the regulation of DNA transcription and cell differentiation, in primary cilia morphogenesis, and with several intracellular signaling cascades (such as the ERK/MAP kinase and the G-protein related responses). These results provide new insights about the possible genetic mechanisms underlying adipocyte differentiation and IMF deposition in Italian Large White pigs intended for the production of PDO hams. The identified new molecular processes supply a set of candidate genes for further detection of genetic polymorphisms associated with changes in the expression level of the DEGs and involved in IMF deposition. Further studies are needed to explore the drivers of this complex process and find possible molecular markers useful for the selection of pigs with improved meat quality features.

## Supporting information

S1 Fig Scale independence and mean connectivity parameters for the different values of Soft Threshold power. The red line cuts the graph in correspondence of the highest R^2^ for the Scale Free Topology Model and thus the first value of the Soft Threshold power located above the red line is the parameter chosen to obtain a better representation of the weighted gene co-expression network.
(TIF)

S1 File Results of the weighted correlation analysis between the expressed genes and the intramuscular fat (IMF) content performed with the WGCNA package. Table 1: the module significance (the overall correlation between the expression of the genes significantly included in each module and IMF content) and the relative *P*-values. Table 2: the genes membership in each of the identified modules (highest and lowest values indicate genes with a high weight in the module eigengene). Table 3: the *P*-values of the genes membership in each of the identified modules. Table 4: the overall genes significance (GS) for the IMF trait (GS is an aggregate measure that indicates the importance of the gene for the IMF deposition; the highest or the lowest are the values of GS and the more the gene is biologically significant for the trait) and the relative *P*-values.
(XLSX)

S1 Table Sequence, amplicon length and annealing temperatures (TM) for each primer couple used for the validation with RT-qPCR. (DOCX)

S2 Table Mapping statistics with the percentages of reads. For each sample are reported the percentages of the uniquely mapped reads, multi-reads and unmapped reads against the *Sus scrofa* reference genome 11.1.
(DOCX)

S3 Table The list of the genes found differentially expressed (DE) with q value ≤ 0.10. (DOCX)

S4 Table ClueGO Gene Ontology (GO) results of the genes in the four significant modules obtained from WGCNA. (XLSX)
