# Supplementary figures and images for: Muscle transcriptome analysis identifies genes involved in ciliogenesis and the molecular cascade associated with intramuscular fat content in Large White heavy pigs
Source: PLoS One. 2020 May 19;15(5):e0233372. doi: 10.1371/journal.pone.0233372 (PMC7237010; doi:10.1371/journal.pone.0233372)

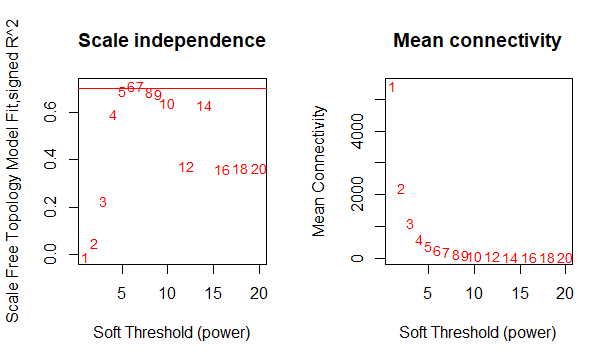

Supplement: S1 Fig — The red line cuts the graph in correspondence of the highest R2 for the Scale Free Topology Model and thus the first value of the Soft Threshold power located above the red line is the parameter chosen to obtain a better representation of the weighted gene co-expression network. (TIF) [file pone.0233372.s001.tif]
