# Supplementary material for: Muscle transcriptome analysis identifies genes involved in ciliogenesis and the molecular cascade associated with intramuscular fat content in Large White heavy pigs
Source: PLoS One. 2020 May 19;15(5):e0233372. doi: 10.1371/journal.pone.0233372 (PMC7237010; doi:10.1371/journal.pone.0233372)
Supplement: S1 Table — (DOCX) [file pone.0233372.s003.docx]

**S1 Table. Sequence, amplicon length and annealing temperatures (TM) for each primer couple used for the validation with RT-qPCR.**

| **Gene name** | **Acronym** | **Accession number** | **Primers** | **TM (°C)** | **Amplicon Length (bp)** |
| --- | --- | --- | --- | --- | --- |
| **Target genes** | | | | | |
| *DnaJ Heat Shock Protein Family (Hsp40) Member B1* | *DNAJB1* | ENSSSCT00000015061.3 transcript sequence | F: 5’- ACCCGCACTTTAACCTGTTG-3’ | 66 | 93 |
|  |  |  | R: 5’- AGGGAACCAATAGCGACCTT-3’ |  |  |
| *Lebercilin 5* | *LCA5* | XM_021092140.1 | F: 5’- AGGCTTCAGTACAGGCAGGA-3’ | 66 | 143 |
|  |  |  | R: 5’- GTTGCCCGTTCTTTCTCTTG-3’ |  |  |
| *LIM Domain Kinase 1* | *LIMK1* | XM_021086335.1 | F: 5’- AGAGCACTCCCACACTGTCC-3’ | 60 | 108 |
|  |  |  | R: 5’- GTGCCATTGATTTCCAGGAT-3’ |  |  |
| *Peroxisome Proliferator Activated*  *Receptor Alpha* | *PPARA* | NM_001044526.1 | F: 5’-CTTGGACTTGAACGACCAGG-3’ | 60 | 96 |
|  |  |  | R: 5’-TCCCGTCCTTGTTCATCACA-3’ |  |  |
| *Transforming Acidic Coiled-coil Containing Protein 2* | *TACC2* | [NM_001258352.1](https://www-ncbi-nlm-nih-gov.ezproxy.unibo.it/entrez/viewer.fcgi?db=nucleotide&id=385275111) | F: 5’- GCCCCTCAAGACTGACACAT-3’ | 66 | 77 |
|  |  |  | R: 5’- GGTCCTGAGAAGGTGGATCA-3’ |  |  |
| **Normalizing genes** | | | | | |
| *Beta-2-Microglobulin* | *B2M* | NM_213978.1 | F: 5’-CCTTCTGGTCCACACTGAGT-3’ | 66 | 99 |
|  |  |  | R: 5’-TCCCACTTAACTATCTTGGGCT-3’ |  |  |
| *Ribosomal Protein L32* | *RPL32* | NM_001001636.1 | F: 5’-TCATGGCTGCTCTCAGACCC-3’ | 63 | 145 |
|  |  |  | R: 5’-CTTCTCCGCACCCTGTTGT-3’ |  |  |
| *Ribosomal Protein S18* | *RPS18* | NM_213940.1 | F: 5’-CATGTGGTGTTGAGGAAAGCA-3’ | 63 | 105 |
|  |  |  | R: 5’-TTGGCGAGGATTCTGCATAAT-3’ |  |  |
