# Supplementary material for: Muscle transcriptome analysis identifies genes involved in ciliogenesis and the molecular cascade associated with intramuscular fat content in Large White heavy pigs
Source: PLoS One. 2020 May 19;15(5):e0233372. doi: 10.1371/journal.pone.0233372 (PMC7237010; doi:10.1371/journal.pone.0233372)
Supplement: S2 Table — For each sample are reported the percentages of the uniquely mapped reads, multi-reads and unmapped reads against the Sus scrofa reference genome 11.1. (DOCX) [file pone.0233372.s004.docx]

**S2 Table. Mapping statistics with the percentages of reads.** For each sample are reported the percentages of the uniquely mapped reads, multi-reads and unmapped reads against the *Sus scrofa* reference genome 11.1.

| **Sample** | **Input-reads** | **Unique reads** | **Multi-reads** | **Unmapped reads** |
| --- | --- | --- | --- | --- |
| 1 | 99,232,040 | 84.3% | 9.7% | 6.1% |
| 2 | 94,139,057 | 84.7% | 8.6% | 6.8% |
| 3 | 93,754,402 | 84.1% | 8.1% | 7.8% |
| 4 | 92,805,694 | 83.4% | 8.5% | 8.1% |
| 5 | 89,252,599 | 83.7% | 8.1% | 8.2% |
| 6 | 90,822,452 | 83.4% | 8.3% | 8.3% |
| 7 | 87,838,001 | 79.1% | 11.2% | 9.7% |
| 8 | 95,884,589 | 84.6% | 7.6% | 7.8% |
| 9 | 98,965,214 | 84.9% | 7.9% | 7.2% |
| 10 | 109,316,067 | 84.1% | 8.7% | 7.3% |
| 11 | 104,975,827 | 85.0% | 7.6% | 7.4% |
| 12 | 98,039,492 | 85.7% | 7.7% | 6.6% |
