# Supplementary material for: Muscle transcriptome analysis identifies genes involved in ciliogenesis and the molecular cascade associated with intramuscular fat content in Large White heavy pigs
Source: PLoS One. 2020 May 19;15(5):e0233372. doi: 10.1371/journal.pone.0233372 (PMC7237010; doi:10.1371/journal.pone.0233372)
Supplement: S3 Table — (DOCX) [file pone.0233372.s005.docx]

**S3 Table. The list of the genes found differentially expressed (DE) with q value ≤ 0.10.**

| **ENSEMBL Gene ID** | **Official gene symbol** | **Average expression** | **Log_2_(FC)^a^** | **Log_2_(FC) S.E.^b^** | **Wald statistic** | ***P*-value** | **Adjusted *P*-value (q value)** |
| --- | --- | --- | --- | --- | --- | --- | --- |
| ENSSSCG00000036462 | *RF01848* | 2,175.23 | 9.29 | 1.25 | 7.41 | 1.31E-13 | 1.59E-09 |
| ENSSSCG00000035039 | *RF00016* | 218.04 | -2.03 | 0.33 | -6.11 | 1.01E-09 | 6.15E-06 |
| ENSSSCG00000012026 | *ADAMTS1* | 4,967.48 | -0.91 | 0.16 | -5.85 | 4.91E-09 | 1.99E-05 |
| ENSSSCG00000039904 | *RF00016* | 270.96 | -1.89 | 0.34 | -5.58 | 2.47E-08 | 6.01E-05 |
| ENSSSCG00000031725 | *RF00016* | 319.62 | -1.96 | 0.35 | -5.58 | 2.42E-08 | 6.01E-05 |
| ENSSSCG00000012173 | *SAT1* | 3,576.98 | -1.35 | 0.25 | -5.34 | 9.39E-08 | 1.90E-04 |
| ENSSSCG00000037241 | *RGS2* | 1,663.86 | -1.68 | 0.32 | -5.26 | 1.42E-07 | 2.47E-04 |
| ENSSSCG00000036787 | *APOLD1* | 1,104.76 | -1.40 | 0.27 | -5.24 | 1.64E-07 | 2.49E-04 |
| ENSSSCG00000031429 | *YAP1* | 9,345.37 | -0.69 | 0.14 | -5.02 | 5.10E-07 | 6.19E-04 |
| ENSSSCG00000037608 | *LRRC70* | 182.03 | -1.06 | 0.21 | -5.04 | 4.63E-07 | 6.19E-04 |
| ENSSSCG00000006105 | *GEM* | 889.15 | -0.79 | 0.16 | -4.89 | 9.89E-07 | 1.00E-03 |
| ENSSSCG00000013784 | *DNAJB1* | 10,901.19 | -1.60 | 0.33 | -4.86 | 1.19E-06 | 1.11E-03 |
| ENSSSCG00000040160 | *MMP24* | 689.48 | 0.60 | 0.13 | 4.78 | 1.76E-06 | 1.53E-03 |
| ENSSSCG00000032962 | *ZNF664* | 3,930.92 | 0.96 | 0.20 | 4.71 | 2.51E-06 | 1.97E-03 |
| ENSSSCG00000016925 | *PLK2* | 2,397.77 | -1.34 | 0.29 | -4.67 | 2.96E-06 | 2.08E-03 |
| ENSSSCG00000030378 | *LIMK1* | 7,740.40 | 0.73 | 0.16 | 4.59 | 4.45E-06 | 2.58E-03 |
| ENSSSCG00000036832 | *STK40* | 8,961.08 | 0.76 | 0.17 | 4.53 | 5.95E-06 | 3.03E-03 |
| ENSSSCG00000016929 | *PDE4D* | 20,370.08 | 0.68 | 0.15 | 4.53 | 5.99E-06 | 3.03E-03 |
| ENSSSCG00000000006 | *PPARA* | 1,547.78 | 0.64 | 0.14 | 4.49 | 7.25E-06 | 3.52E-03 |
| ENSSSCG00000026392 | *BSDC1* | 5,798.78 | 0.63 | 0.14 | 4.43 | 9.48E-06 | 4.43E-03 |
| ENSSSCG00000030632 | *UBE2V1* | 3,965.36 | -1.81 | 0.41 | -4.42 | 9.96E-06 | 4.48E-03 |
| ENSSSCG00000024312 | *ID4* | 910.67 | -1.45 | 0.33 | -4.40 | 1.07E-05 | 4.52E-03 |
| ENSSSCG00000034207 | *CEBPB* | 8,883.82 | 0.84 | 0.19 | 4.32 | 1.59E-05 | 6.24E-03 |
| ENSSSCG00000034044 | *RASD1* | 467.54 | -1.20 | 0.28 | -4.30 | 1.68E-05 | 6.38E-03 |
| ENSSSCG00000012276 | *SYN1* | 806.36 | 0.64 | 0.15 | 4.28 | 1.88E-05 | 6.71E-03 |
| ENSSSCG00000004663 | *SEMA6D* | 717.06 | -0.69 | 0.16 | -4.28 | 1.87E-05 | 6.71E-03 |
| ENSSSCG00000016095 | *CLK1* | 6,231.13 | -0.91 | 0.21 | -4.27 | 1.95E-05 | 6.76E-03 |
| ENSSSCG00000006940 | *CCN1* | 4,916.65 | -1.14 | 0.27 | -4.21 | 2.57E-05 | 8.21E-03 |
| ENSSSCG00000028078 | *DPH1* | 1,022.09 | -0.60 | 0.14 | -4.17 | 3.11E-05 | 9.69E-03 |
| ENSSSCG00000028063 | *TACC2* | 5,354.06 | 0.61 | 0.15 | 4.12 | 3.84E-05 | 1.14E-02 |
| ENSSSCG00000032416 | *RGS1* | 495.97 | -1.44 | 0.35 | -4.08 | 4.43E-05 | 1.28E-02 |
| ENSSSCG00000014960 | *AMOTL1* | 18,792.95 | 0.67 | 0.16 | 4.07 | 4.72E-05 | 1.30E-02 |
| ENSSSCG00000016882 | *PARP8* | 361.84 | -0.84 | 0.21 | -4.06 | 4.83E-05 | 1.30E-02 |
| ENSSSCG00000022925 | *SLC2A3* | 2,646.43 | -1.54 | 0.38 | -4.07 | 4.75E-05 | 1.30E-02 |
| ENSSSCG00000008645 | *ID2* | 731.24 | -0.91 | 0.23 | -4.00 | 6.35E-05 | 1.61E-02 |
| ENSSSCG00000036893 | *PTHLH* | 86.11 | -1.03 | 0.26 | -3.99 | 6.50E-05 | 1.61E-02 |
| ENSSSCG00000021411 | *HIST1H2BD* | 1,041.72 | 1.76 | 0.44 | 3.96 | 7.44E-05 | 1.74E-02 |
| ENSSSCG00000036213 | *FGF2* | 1,158.76 | -0.75 | 0.19 | -3.96 | 7.59E-05 | 1.74E-02 |
| ENSSSCG00000032749 | *PCDH18* | 1,054.30 | -0.69 | 0.18 | -3.94 | 8.06E-05 | 1.81E-02 |
| ENSSSCG00000004469 | *LCA5* | 230.42 | -1.26 | 0.32 | -3.93 | 8.65E-05 | 1.88E-02 |
| ENSSSCG00000009517 | *GPR183* | 142.76 | -1.47 | 0.38 | -3.86 | 1.12E-04 | 2.30E-02 |
| ENSSSCG00000012956 | *PACS1* | 774.03 | 0.62 | 0.16 | 3.85 | 1.20E-04 | 2.42E-02 |
| ENSSSCG00000038149 | *KCNE4* | 132.97 | -1.19 | 0.31 | -3.80 | 1.43E-04 | 2.78E-02 |
| ENSSSCG00000029753 | *CYTIP* | 600.18 | -1.04 | 0.28 | -3.75 | 1.76E-04 | 3.14E-02 |
| ENSSSCG00000005599 | *RABEPK* | 322.10 | -0.68 | 0.18 | -3.74 | 1.82E-04 | 3.20E-02 |
| ENSSSCG00000010626 | *RBM20* | 5,520.03 | 0.85 | 0.23 | 3.73 | 1.94E-04 | 3.37E-02 |
| ENSSSCG00000026686 | *PDZD9* | 2,816.34 | 1.06 | 0.29 | 3.69 | 2.23E-04 | 3.43E-02 |
| ENSSSCG00000026733 | *HIPK2* | 1,736.76 | 0.71 | 0.19 | 3.69 | 2.21E-04 | 3.43E-02 |
| ENSSSCG00000001247 | *ZFP57* | 65.40 | -1.23 | 0.33 | -3.70 | 2.14E-04 | 3.43E-02 |
| ENSSSCG00000008959 | *CXCL2* | 553.58 | -1.50 | 0.41 | -3.69 | 2.21E-04 | 3.43E-02 |
| ENSSSCG00000016956 | *MAST4* | 11,030.05 | 0.80 | 0.22 | 3.68 | 2.32E-04 | 3.53E-02 |
| ENSSSCG00000031763 | *LOC100524118 - somatomedin-B and thrombospondin type-1 domain-containing protein-like* | 353.30 | 1.48 | 0.40 | 3.66 | 2.53E-04 | 3.70E-02 |
| ENSSSCG00000011516 | *EIF4E3* | 1,648.25 | -1.06 | 0.29 | -3.63 | 2.80E-04 | 3.96E-02 |
| ENSSSCG00000038965 | *ARC* | 643.04 | -3.10 | 0.86 | -3.62 | 2.94E-04 | 4.01E-02 |
| ENSSSCG00000002281 | *FNTB* | 3,550.28 | 0.69 | 0.19 | 3.61 | 3.09E-04 | 4.08E-02 |
| ENSSSCG00000020970 | *IL6* | 541.64 | -4.03 | 1.12 | -3.60 | 3.20E-04 | 4.18E-02 |
| ENSSSCG00000032967 | *CACNB3* | 263.91 | 1.61 | 0.45 | 3.55 | 3.82E-04 | 4.78E-02 |
| ENSSSCG00000039651 | *SLC2A5* | 414.03 | -1.62 | 0.46 | -3.55 | 3.92E-04 | 4.85E-02 |
| ENSSSCG00000026454 | *PMAIP1* | 374.47 | -1.33 | 0.38 | -3.53 | 4.14E-04 | 5.08E-02 |
| ENSSSCG00000019418 | *SNORA4* | 151.91 | -0.82 | 0.23 | -3.53 | 4.22E-04 | 5.12E-02 |
| ENSSSCG00000020912 | *HECTD2* | 850.97 | -0.59 | 0.17 | -3.50 | 4.62E-04 | 5.46E-02 |
| ENSSSCG00000023716 | *TNFAIP6* | 668.73 | -1.63 | 0.47 | -3.50 | 4.63E-04 | 5.46E-02 |
| ENSSSCG00000008624 | *LPIN1* | 7,784.18 | 0.72 | 0.21 | 3.48 | 4.93E-04 | 5.64E-02 |
| ENSSSCG00000006288 | *SELP* | 548.31 | -1.09 | 0.31 | -3.48 | 5.07E-04 | 5.73E-02 |
| ENSSSCG00000027660 | *IFI44L* | 737.76 | -0.72 | 0.21 | -3.45 | 5.62E-04 | 5.84E-02 |
| ENSSSCG00000011877 | *CD86* | 390.27 | -0.97 | 0.28 | -3.46 | 5.40E-04 | 5.84E-02 |
| ENSSSCG00000008535 | *CLIP4* | 2,205.02 | -1.52 | 0.44 | -3.46 | 5.49E-04 | 5.84E-02 |
| ENSSSCG00000011102 | *NRP1* | 3,781.37 | 0.71 | 0.21 | 3.43 | 5.95E-04 | 5.87E-02 |
| ENSSSCG00000007941 | *CDIP1* | 2,660.49 | 0.61 | 0.18 | 3.40 | 6.70E-04 | 6.36E-02 |
| ENSSSCG00000039272 | *IP6K3* | 6,859.82 | 0.74 | 0.22 | 3.38 | 7.26E-04 | 6.83E-02 |
| ENSSSCG00000040575 | *ISG15* | 291.92 | -0.74 | 0.22 | -3.37 | 7.59E-04 | 7.09E-02 |
| ENSSSCG00000009794 | *MLXIP* | 18,288.35 | 0.70 | 0.21 | 3.35 | 8.14E-04 | 7.22E-02 |
| ENSSSCG00000037910 | *DUSP6* | 1,379.19 | -0.71 | 0.21 | -3.35 | 8.21E-04 | 7.22E-02 |
| ENSSSCG00000015893 | *SLC4A10* | 1,111.37 | -1.33 | 0.40 | -3.34 | 8.36E-04 | 7.26E-02 |
| ENSSSCG00000003789 | *CTH* | 423.77 | -0.73 | 0.22 | -3.33 | 8.74E-04 | 7.47E-02 |
| ENSSSCG00000011570 | *IRAK2* | 476.97 | -0.71 | 0.21 | -3.31 | 9.32E-04 | 7.92E-02 |
| ENSSSCG00000006311 | *DUSP27* | 9,813.64 | -1.41 | 0.43 | -3.31 | 9.49E-04 | 8.00E-02 |
| ENSSSCG00000035667 | *KBTBD13* | 222.10 | -0.70 | 0.21 | -3.28 | 1.02E-03 | 8.33E-02 |
| ENSSSCG00000011672 | *RASA2* | 946.06 | -0.60 | 0.18 | -3.26 | 1.12E-03 | 8.91E-02 |
| ENSSSCG00000004573 | *C2CD4A* | 64.77 | 1.20 | 0.37 | 3.24 | 1.18E-03 | 9.12E-02 |
| ENSSSCG00000002135 | *PNP* | 411.12 | -1.27 | 0.39 | -3.24 | 1.18E-03 | 9.12E-02 |
| ENSSSCG00000040751 (ENSSSCG00000044553) | *DDIT3* | 5,986.90 | -1.09 | 0.34 | -3.24 | 1.20E-03 | 9.14E-02 |
| ENSSSCG00000024045 | *CASTOR2* | 8,020.85 | 0.63 | 0.20 | 3.23 | 1.25E-03 | 9.49E-02 |
| ENSSSCG00000010203 | *HNRNPF* | 10,240.87 | 0.84 | 0.26 | 3.22 | 1.26E-03 | 9.50E-02 |
| ENSSSCG00000000234 | *TAMALIN* | 227.33 | -0.89 | 0.28 | -3.21 | 1.34E-03 | 9.84E-02 |
| ENSSSCG00000012076 | *MX2* | 730.91 | -1.03 | 0.32 | -3.20 | 1.36E-03 | 9.85E-02 |
| ENSSSCG00000012993 | *SLC25A45* | 451.30 | 0.74 | 0.23 | 3.19 | 1.45E-03 | 1.00E-01 |
| ENSSSCG00000014060 | *KIAA1191* | 1,929.34 | 0.63 | 0.20 | 3.19 | 1.42E-03 | 1.00E-01 |
| ENSSSCG00000032464 | *PURB* | 340.46 | 0.63 | 0.20 | 3.18 | 1.45E-03 | 1.00E-01 |

^a^ log_2_(Fold Change) of the gene expression levels in the low IMF group *vs.* the high IMF group.

^b^ Standard error (S.E.) of the log_2_(Fold Change)
